# Loschmidt echo for deformed Wigner matrices

**DOI:** 10.1007/s11005-025-01904-5

**Published:** 2025-01-30

**Authors:** László Erdős, Joscha Henheik, Oleksii Kolupaiev

**Affiliations:** Institute of Science and Technology Austria, Am Campus 1, 3400 Klosterneuburg, Austria

**Keywords:** Quantum dynamics, Loschmidt echo, Matrix Dyson equation, 60B20, 82C10

## Abstract

We consider two Hamiltonians that are close to each other, $H_1 \approx H_2$, and analyze the time decay of the corresponding *Loschmidt echo* $\mathfrak {M}(t):= |\langle \psi _0, \textrm{e}^{\textrm{i} t H_2} \textrm{e}^{-\textrm{i} t H_1} \psi _0 \rangle |^2$ that expresses the effect of an imperfect time reversal on the initial state $\psi _0$. Our model Hamiltonians are deformed Wigner matrices that do not share a common eigenbasis. The main tools are new two-resolvent laws for such $H_1$ and $H_2$.

## Introduction

Recent quantum technological advances put quantum mechanical time reversal procedures in the focus of both experimental [25, 32, 37, 38, 40, 42] and theoretical [16, 17, 30, 31, 33, 43–45] research (see also the review [29] for a concise overview). The basic physical setup consists of an initial (normalized) quantum state $\psi _0$ and two self-adjoint Hamiltonians close to each other, $H_1 \approx H_2$, each governing the evolution of the system during a time span *t*. First, the initial state $\psi _0$ evolves under the Hamiltonian $H_1$ from time zero to *t*, resulting in the state $\psi _t = \exp {(-\textrm{i}H_1 t)} \psi _0$. Then, during a second evolution between *t* and 2*t*, one applies the Hamiltonian $H_2$ backward in time, equivalently the Hamiltonian $-H_2$ in forward time, aiming to recover the initial state $\psi _0$. A schematic summary of this process is given by

1.1 $$\begin{aligned} \psi _0 \xrightarrow [~~~H_1~~~]{t} \psi _t \xrightarrow [~~-H_2~~]{t} \psi _0' \,. \end{aligned}$$

Note that if $H_2 = H_1$, the restoration of $\psi _0$ would be perfect, $\psi _0' =\psi _0$ for any time *t*. However, in realistic setup the second Hamiltonian is never a perfect copy of the first one: the nonzero difference between $H_1$ and $H_2$ regularly leads to an imperfect recovery $\psi _0'$ of $\psi _0$ and the discrepancy also depends on time.

This imperfection in the time reversal is captured in the scalar *overlap function* [28, 45, 49] (sometimes also called *fidelity amplitude* [27, 28, 50])

1.2 $$\begin{aligned} \mathfrak {S}(t) = \mathfrak {S}_{H_1, H_2}^{(E_0)}(t) := \big \langle \psi _0, \textrm{e}^{\textrm{i}H_2 t} \textrm{e}^{-\textrm{i}H_1 t} \psi _0 \big \rangle \end{aligned}$$

where it is assumed that the initial state is supported^1^ around its energy $\langle \psi _0, H_1 \psi _0 \rangle \approx \langle \psi _0, H_2 \psi _0 \rangle \approx E_0$. The central object of our paper is the absolute value square of the overlap function

1.3 $$\begin{aligned} \mathfrak {M}(t) = \mathfrak {M}_{H_1, H_2}^{(E_0)}(t) := \left| \mathfrak {S}_{H_1, H_2}^{(E_0)}(t) \right| ^2\,. \end{aligned}$$

This was coined the *fidelity*, e.g., by Gorin et al. [28], or the *Loschmidt echo* by Peres [41] and Jalabert–Pastawski in [34] owing to its connection to the classical Loschmidt’s paradox of time reversibility [6, 39].

In addition to (1.2)–(1.3), we will also consider an *averaged overlap function* and an *averaged Loschmidt echo*, defined as

1.4 $$\begin{aligned} \overline{\mathfrak {S}}(t) = \overline{\mathfrak {S}}_{H_1, H_2}^{(E_0, \eta _0)}(t) := \textrm{Av}\big [ \mathfrak {S}_{H_1, H_2}^{(E)}(t)\big ] \quad \,{\text {and}}\, \quad \overline{\mathfrak {M}}(t) = \overline{\mathfrak {M}}_{H_1, H_2}^{(E_0, \eta _0)}(t):= \left| \overline{\mathfrak {S}}_{H_1, H_2}^{(E_0, \eta _0 )}(t) \right| ^2, \end{aligned}$$

respectively. In (1.4), by $\textrm{Av}[...]$, we denoted an averaging over initial states with energies *E* in a small energy window of size $\eta _0$ around $E_0$ (see (2.5) below for a precise implementation of this concept).

**Fig. 1:**
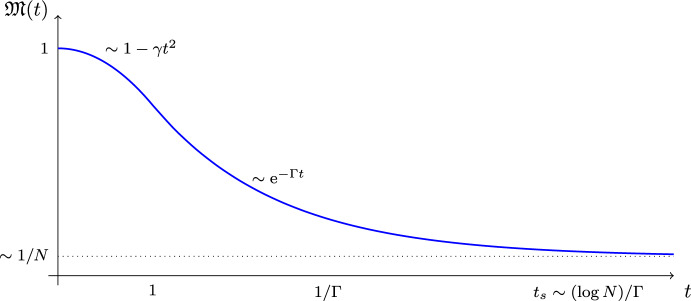
Illustrated is the typical behavior of the Loschmidt echo in its three consecutive phases: Short-time parabolic decay, intermediate-time asymptotic decay, and long-time saturation. In both of our main results (1.6)–(1.7), the decay parameters $\gamma$ and $\Gamma$ generally satisfy $\gamma \sim \Gamma \sim N^{-1}\textrm{Tr}(H_1-H_2)^2$; cf. (1.8)

The Loschmidt echo is a basic object in the study of complex quantum system and has attracted considerable attention in different areas of research, e.g., quantum chaos [30, 31, 33, 34, 41, 45], quantum information theory [24, 26], and statistical mechanics [16, 17, 43, 44]. The Loschmidt echo, as a measurable physical quantity, is observed and predicted to follow a quite universal behavior as a function of time (cf. the discussion of our main results around (1.6)–(1.7) below). On a high level (see [29]), the reason for the robust universal features is that the subsequent forward and backward evolutions act as a “filter" for irrelevant details. The typical behavior of the Loschmidt echo can be structured in three consecutive phases (see Fig. 1, cf. also [29, Figure 4]): After an initial short-time parabolic decay, $\mathfrak {M}(t) \approx 1 - \gamma t^2$, the Loschmidt echo exhibits an intermediate-time asymptotic exponential decay^2^, $\mathfrak {M}(t) \approx \textrm{e}^{-\Gamma t}$. Finally, at times *t* beyond the so-called *saturation time* $t_s \sim (\log N)/\Gamma$, where *N* is the (effective) Hilbert space dimension, it saturates at a value inversely proportional to *N*, i.e., $\mathfrak {M}(t) \sim 1/N$. We restrict our study to the first two regimes. For the explanation of technical issues related to the third regime, we refer to the discussion below Theorem 2.4.

There are several ways to determine the behavior of the Loschmidt echo in a given system (see the review [29]): One standard option is to employ semi-classical approximations [34, 49, 52], another one is numerical evaluation [19, 47, 48]. Here, following E. Wigner’s original vision of describing chaotic quantum systems by large random matrices [51] and the Bohigas–Giannoni–Schmit (BGS) conjecture [5] (see also further extensive physics literature [7, 8, 15, 17, 18, 27, 35]), we model (part of) the Hamiltonian(s) $H_1, H_2$ by Wigner random matrices with independent entries. In this setup, we can give a mathematically rigorous and quite precise analysis of certain features of the Loschmidt echo; some of them have been predicted in the physics literature.

Before defining the precise model, we first discuss where the name *echo* for $\mathfrak {M}(t)$ comes from. Fix any time $t>0$ and consider the two-step process (1.1). For $s\in [0,2t]$ denote the state at the intermediate time *s* by $\psi _s$, namely, $\psi _s = \textrm{e}^{-\textrm{i}sH_1}\psi _0$ for $s\in [0,t]$ and $\psi _s=\textrm{e}^{\textrm{i}(t-s)H_2}\textrm{e}^{-\textrm{i}t H_1}\psi _0$ for $s\in [t,2t]$. Comparing this notation to (1.1) we see that $\psi _{2t}=\psi _0'$. Denote further the (squared) overlap of $\psi _0$ and $\psi _s$ by

1.5 $$\begin{aligned} \mathfrak {P}_t(s):=\vert \langle \psi _0,\psi _s\rangle \vert ^2. \end{aligned}$$

**Fig. 2:**
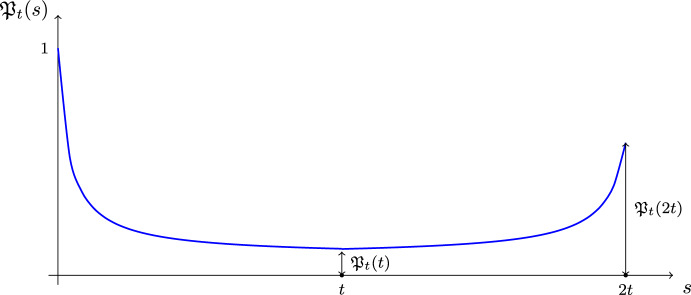
[*Echo feature*] Schematic behavior of the overlap $\mathfrak {P}_t(s)$ from (1.5) for $s\in [0,2t]$. At the midpoint, $s=t$, typically $\mathfrak {P}_t(t) \ll \mathfrak {P}_t(2t)$, which indicates a partial recovery between time *t* and 2*t* of the original complete overlap at time $s=0$

This quantity depends also on $\psi _0$ and $H_1, H_2$, but we suppress this dependence in notations for simplicity. We call $\mathfrak {P}_t(s)$, $s\in [0,2t]$, the *Loschmidt echo process*. Clearly, $\mathfrak {P}_t(0)=1$ and $\mathfrak {P}_t(2t)=\mathfrak {M}(t)$. Later in Corollary 2.5 we show that $\overline{\mathfrak {P}}_t(t)\ll \overline{\mathfrak {P}}_t(2t)$ under suitable assumptions, where $\overline{\mathfrak {P}}_t$ is an averaged version of $\mathfrak {P}_t$ defined in (2.9a)-(2.9b). This result means that typically the original complete overlap $\mathfrak {P}_t(0)=1$ is partially recovered at the final moment of time 2*t*, though at the intermediate time *t* it is much smaller than $\mathfrak {P}_t(2t)$ (see Fig. 2).

As our main result, we rigorously prove the decay of the Loschmidt echo for two different physical settings (called *Scenario I* and *Scenario II*), which we now describe somewhat informally (see Sect. 2 for more precise statements containing all the technical details).

For our first result (Scenario I, Theorem 2.4), we consider two *deformed* $N\times N$ *Wigner matrices* $H_j = D_j + W$, $j =1,2$, with bounded deterministic $D_j$, satisfying $D_1 \approx D_2$, and *W* a (common) random Wigner matrix. This setup corresponds to an arbitrary deterministic system modeled by the Hamiltonian $D_1$ and the time reversed Hamiltonian $D_2$ nearby, which are both subject to an overall mean-field noise described by the same Wigner matrix *W* throughout the whole echo process. In this setting, for an energy $E_0$ in the bulk of the density of states of both $H_1$ and $H_2$, we consider the averaged Loschmidt echo (1.4). Our result in Theorem 2.4 then shows (i) *short-time parabolic decay* and (ii) *intermediate-time asymptotic decay* of the form

1.6 $$\begin{aligned} \overline{\mathfrak {M}}(t) \approx {\left\{ \begin{array}{ll} 1 - \gamma t^2 \quad & {\text {for}} \quad t \ll 1 \\ \textrm{e}^{- \Gamma t} \quad & {\text {for}} \quad 1 \ll t \lesssim \Delta ^{-2}. \end{array}\right. } \end{aligned}$$

Both *decay parameters* satisfy $\gamma \sim \Delta ^2$ and $\Gamma \sim \Delta ^2$, where $\Delta := \langle (D_1 -D_2)^2 \rangle ^{1/2}$, and depend on $E_0$ and the density of states at $E_0$. Here, we introduced the notation $\langle A\rangle :=\frac{1}{N}\textrm{Tr}\, A$ for any $N\times N$ matrix *A*. We point out that the quadratic relation $\Gamma \sim \Delta ^2$ is in perfect agreement with *Fermi’s golden rule*.

For our second main result (Scenario II, Theorem 2.10), we consider a physically different situation: Now the two Hamiltonians^3^ are $H_1 = D$ and $H_2 = D + \lambda W$ with the same deterministic *D*, a standard Wigner matrix *W* and a small parameter $|\lambda | \ll 1$. The normalization is chosen such that $\textbf{E}\langle W^2\rangle =1$. Hence, the imperfection along the backward evolution is modeled by a small Wigner matrix $\lambda W$ indicating an additive noise (see, e.g., [16, Eq. (31)] or [17, Eq. (1)]). For a normalized initial state $\psi _0 \in \textbf{C}^N$ supported in the bulk of the density of states of both $H_1$ and $H_2$ with energy $\langle \psi _0, H_1 \psi _0 \rangle \approx \langle \psi _0, H_2 \psi _0 \rangle \approx E_0$, we now consider the usual Loschmidt echo (1.3) without averaging. Similarly to (1.6), our result in Theorem 2.10 then shows (i) *short-time parabolic decay* and (ii) *intermediate-time asymptotic decay* of the form

1.7 $$\begin{aligned} \mathfrak {M}(t) \approx {\left\{ \begin{array}{ll} 1 - \gamma t^2 \quad & {\text {for}} \quad t \ll 1 \\ \textrm{e}^{- \Gamma t} \quad & {\text {for}} \quad 1 \ll t \lesssim \lambda ^{-2}. \end{array}\right. } \end{aligned}$$

Here the *decay parameters* satisfy $\gamma = \lambda ^2$ and $\Gamma = 2 \pi \rho _0(E_0) \lambda ^2$, where $\rho _0$ is the (limiting, as $N\rightarrow \infty$) density of states of *D*. Finally, we note that since $\textbf{E}\langle W^2 \rangle = 1$, in both of our scenarios (1.6)–(1.7) the decay parameters $\gamma$ and $\Gamma$ satisfy the general relation

1.8 $$\begin{aligned} \gamma \sim \Gamma \sim \textbf{E}\langle (H_1 -H_2)^2 \rangle \,. \end{aligned}$$

As corollaries to our main results (1.6)–(1.7) in Theorems 2.4 and 2.10, we also consider the *scrambled Loschmidt echo* [16, 36, 44] $\mathfrak {M}_\delta ^{\mathrm sc}(t)$ and its averaged analog $\overline{\mathfrak {M}}_\delta ^{\mathrm sc}(t)$. They are defined from

1.9 $$\begin{aligned} \mathfrak {S}_\delta ^{\textrm{sc}}(t) := \big \langle \psi _0, \textrm{e}^{\textrm{i}H_2 t} \textrm{e}^{-\textrm{i}\delta V}\textrm{e}^{-\textrm{i}H_1 t} \psi _0 \big \rangle \end{aligned}$$

and its averaged analog $\overline{\mathfrak {S}}_\delta ^{\textrm{sc}}(t)$ as

$$ \mathfrak {M}_\delta ^{\mathrm sc}(t): = \big | \mathfrak {S}_\delta ^{\textrm{sc}}(t) \big |^2 \quad {\text {and}} \quad \overline{\mathfrak {M}}_\delta ^{\mathrm sc}(t): = \big | \overline{\mathfrak {S}}_\delta ^{\textrm{sc}}(t) \big |^2, $$

exactly as in (1.3)–(1.4), respectively. In (1.9), $H_1$ and $H_2$ are the two Hamiltonians either from Scenario I or Scenario II. The idea behind the quantity in (1.9) is that, between the forward and backward evolution, there is a (short) *scrambling time* $\delta$, in which the system is uncontrolled and governed by another self-adjoint *scrambling Hamiltonian* *V* [16]. Similarly to (1.1), a schematic summary of this process is given by

1.10 $$\begin{aligned} \psi _0 \xrightarrow [~~~H_1~~~]{t} \psi _t \xrightarrow [~~~V~~~]{\delta } \psi _t' \xrightarrow [~~-H_2~~]{t} \psi _0' \,. \end{aligned}$$

In Corollaries 2.6 and 2.11 (of Theorems 2.4 and 2.10, respectively), we model the scrambling Hamiltonian by another Wigner matrix, $V:= \widetilde{W}$, that is *independent* of *W*; see [16]. As a result, we find that

1.11 $$\begin{aligned} \overline{\mathfrak {M}}_\delta ^{\mathrm sc}(t) \approx (\varphi (\delta ))^2 \, \overline{\mathfrak {M}}(t) \quad {\text {and}} \quad \mathfrak {M}_\delta ^{\mathrm sc}(t) \approx (\varphi (\delta ))^2 \, \mathfrak {M}(t) \end{aligned}$$

in the setting of Scenario I and Scenario II, respectively, where we denoted $\varphi (\delta ):= J_1(2 \delta )/\delta$ and $J_1$ is the first order Bessel function of the first kind. Note that in (1.11) we see the effects of the scrambling Hamiltonian *V* and the imperfect time reversal of $H_1$ and $H_2$ to completely decouple (cf. [16, Eq. (35)]).

We point out that Scenario II, discussed around (1.7), and the corollaries described in (1.11) are primarily given to provide a more comprehensive view of Loschmidt echoes modeled with Wigner matrices. Technically, these are obtained by simple modifications of earlier results and techniques [10, 22] (see the proof in Sect. 7 for details). The mathematically novel principal part of this work therefore consists of Theorem 2.4 analyzing Scenario I.

The proof of Theorem 2.4 relies on a new *two-resolvent global law*, i.e., a concentration estimate for products of resolvents $G_i(z_i):= (H_i - z_i)^{-1}$ for $z_i \in \textbf{C}{\setminus } \textbf{R}$ as the dimension *N* of the matrix becomes large. By functional calculus, this can then be used for computing more complicated functions of $H_i$, like the exponential, and thus connecting to the time evolutions above. A typical global law computes, e.g.,

1.12 $$\begin{aligned} \langle \psi _0, G_2(z_2) G_1(z_1) \psi _0 \rangle \end{aligned}$$

to leading order in *N* with error terms vanishing like $N^{-1/2+\epsilon }$ with very high probability. The main novelty of this paper is a precise estimate on the deterministic leading term to (1.12). While it is well known that $G_i(z_i) \approx M_i(z_i)$, where the deterministic matrix $M_i(z_i)$ is the solution of the *Matrix Dyson equation* (2.1), it does *not* hold that $G_2(z_2) G_1(z_1) \approx M_2(z_2) M_1(z_1)$ owing to correlations between $G_1$ and $G_2$. The correct approximation is

1.13 $$\begin{aligned} G_2(z_2) G_1(z_1) \approx \frac{M_2(z_2) M_1(z_1)}{1 - \langle M_1(z_1) M_2(z_2)\rangle } \,. \end{aligned}$$

To control (1.13), we hence need to estimate the denominator of (1.13), which is well known in case of $H_1 = H_2$, i.e., $D_1 = D_2$ [9, 13, 22]. Here, however, the analysis of (1.13) is much more intricate, since for general $D_1, D_2$ the deterministic approximations $M_1(z_1), M_2(z_2)$ do *not* commute. In our main Proposition 4.2, we optimally track the dependence of (1.13) on the difference $D_1 - D_2$ of the two deformations and on $z_1-z_2$.

### Notations

For positive quantities *f*, *g* we write $f\lesssim g$ (or $f=\mathcal {O}(g)$) and $f\sim g$ if $f \le C g$ or $c g\le f\le Cg$, respectively, for some constants $c,C>0$ which only depend on the constants appearing in the moment condition (see Assumption 2.1), the bound on *M* in Assumption 2.2, the constants from Assumption 2.8, or the bulk parameter $\kappa$ from (2.3). In informal explanations, we frequently use the notation $f \ll g$, which indicates that *f* is "much smaller" than *g*. Moreover, we shall also write $w \approx z$ to indicate the closeness of $w, z \in \textbf{C}$ with a not precisely specified error.

For any natural number *n*, we set $[n]: =\{ 1, 2,\ldots ,n\}$. Matrix entries are indexed by lowercase Roman letters *a*, *b*, *c*, ... from the beginning of the alphabet. We denote vectors by bold-faced lowercase Roman letters ${\varvec{x}}, {\varvec{y}}\in \textbf{C}^N$, or lower case Greek letters $\psi , \phi \in \textbf{C}^N$, for some $N\in \textbf{N}$. Vector and matrix norms, $\Vert {\varvec{x}}\Vert$ and $\Vert A\Vert$, indicate the usual Euclidean norm and the corresponding induced matrix norm. For any $N\times N$ matrix *A* we use the notation $\langle A\rangle := N^{-1}\textrm{Tr} A$ for its normalized trace and denote the spectrum of *A* by $\sigma (A)$. Moreover, for vectors ${\varvec{x}}, {\varvec{y}}\in \textbf{C}^N$ we denote their scalar product by $\langle {\varvec{x}},{\varvec{y}}\rangle := \sum _{i} \overline{x}_i y_i$. The support of a function *f* is denoted by $\textrm{supp}(f)$.

Finally, we use the concept of “with very high probability” *(w.v.h.p.)* meaning that for any fixed $C>0$, the probability of an *N*-dependent event is bigger than $1-N^{-C}$ for $N\ge N_0(C)$. We also introduce the notion of *stochastic domination* (see, e.g., [20]): given two families of non-negative random variables

$$ X=\left( X^{(N)}(u): N\in \textbf{N}, u\in U^{(N)} \right) \quad \textrm{and}\quad Y=\left( Y^{(N)}(u): N\in \textbf{N}, u\in U^{(N)} \right) $$

indexed by *N* (and possibly some parameter *u* in some parameter space $U^{(N)}$), we say that *X* is stochastically dominated by *Y*, if for all $\xi , C>0$ we have

1.14 $$\begin{aligned} \sup _{u\in U^{(N)}} \textbf{P}\left[ X^{(N)}(u)>N^\xi Y^{(N)}(u)\right] \le N^{-C} \end{aligned}$$

for large enough $N\ge N_0(\xi ,C)$. In this case we use the notation $X\prec Y$ or $X= \mathcal {O}_\prec (Y)$.

## Main results

The key players of our paper are *deformed Wigner matrices*, i.e.,  matrices of the form $H = D + W$, where $D = D^* \in \textbf{C}^{N \times N}$ is a bounded deterministic matrix (called *deformation*), $\Vert D \Vert \le L$ for some *N*-independent $L> 0$ and $W = W^* \in \textbf{C}^{N \times N}$ is a real symmetric or complex Hermitian Wigner matrices. This means, its entries are independently distributed random variables according to the laws^4^$w_{ij} {\mathop {=}\limits ^{\textrm{d}}} N^{-1/2}\chi _{\textrm{od}}$ for $i < j$ and $w_{jj} {\mathop {=}\limits ^{\textrm{d}}} N^{-1/2}\chi _{\textrm{d}}$. For the single entry distributions $\chi _{\textrm{od}}$ and $\chi _{\textrm{d}}$ we assume the following.

### Assumption 2.1

(*Wigner matrix*) We assume that $\chi _{\textrm{d}}$ is a centered real random variable, and $\chi _{\textrm{od}}$ is a real or complex random variable with $\textbf{E}\chi _{\textrm{od}} = 0$ and $\textbf{E}|\chi _{\textrm{od}}|^2 = 1$. Furthermore, we assume the existence of higher moments, namely $\textbf{E}|\chi _{\textrm{d}}|^p + \textbf{E}|\chi _{\textrm{od}}|^p \le C_p$ for all $p\in \textbf{N}$, where $C_p$ are positive constants.

It is well known [2, 21] that the resolvent of *H*, denoted by $G(z):= (H-z)^{-1}$ for $z \in \textbf{C}{\setminus } \textbf{R}$, becomes approximately deterministic in the large *N* limit. Its deterministic approximation (as a matrix) is given by *M*(*z*), the unique solution of the Matrix Dyson equation (MDE)

2.1 $$\begin{aligned} - \frac{1}{M(z)} = z - D + \langle M(z) \rangle \quad {\text {for}} \quad z \in \textbf{C}\setminus \textbf{R}\quad {\text {under the constraint}} \quad \Im z \, \Im M(z) > 0 \,, \end{aligned}$$

where $\Im M(z):= [M(z) - M(z)^*]/2 \textrm{i}$ and positivity is understood as a matrix. The corresponding (*N*-dependent) *self-consistent density of states (scDos)* is defined as

2.2 $$\begin{aligned} \rho (e):=\frac{1}{\pi }\lim _{\eta \downarrow 0}\langle \Im M(e+\textrm{i}\eta )\rangle \,. \end{aligned}$$

This is a compactly supported Hölder-1/3 continuous function on $\textbf{R}$ which is in fact real-analytic on the set $\{ \rho > 0\}$,^5^. The positive harmonic extension of $\rho$ is denoted by $\rho (z):= \pi ^{-1} |\langle \Im M(z) \rangle |$ for $z \in \textbf{C}{\setminus } \textbf{R}$. We point out that not only the tracial quantity $\langle \Im M(e+\textrm{i}\eta )\rangle$ has an extension to the real axis, but the whole matrix $M(e):= \lim _{\eta \downarrow 0} M(e + \textrm{i}\eta )$ is well defined (see Lemma B.1 (b) of the arXiv: 2301.03549 version of [14]). Moreover, for any small $\kappa > 0$ (independent of *N*) we define the $\kappa$-*bulk* of the scDos (2.2) as

2.3 $$\begin{aligned} \textbf{B}_\kappa (\rho ) = \left\{ x \in \textbf{R}\; : \; \rho (x) \ge \kappa \right\} \,. \end{aligned}$$

It is a finite union of disjoint compact intervals, cf. Lemma B.2 in the arXiv: 2301.03549 version of [14]. Note that, for $\Re z \in \textbf{B}_\kappa$ it holds that $\Vert M(z) \Vert \lesssim 1$, as easily follows by taking the imaginary part of (2.1).

Now, the resolvent *G* is close to *M* from (2.1) in the following *averaged* and *isotropic* sense:

2.4 $$\begin{aligned} |\langle (G(z)-M(z))B\rangle |\prec \frac{1}{N|\Im z|}, \qquad |\langle {\varvec{x}}\,, (G(z)-M(z)) {\varvec{y}}\rangle |\prec \frac{1}{\sqrt{N|\Im z|}} \,, \end{aligned}$$

uniformly in deterministic vectors $\Vert {\varvec{x}}\Vert +\Vert {\varvec{y}}\Vert \lesssim 1$ and deterministic matrices $\Vert B\Vert \lesssim 1$. These estimates are called *local laws* when $|\Im z|\ll 1$ and *global laws* when $|\Im z|\gtrsim 1$. To be precise about their validity, we recall that while (2.4) holds for $\Re z \in \textbf{B}_\kappa$ and $\textrm{dist}(\Re z, \textrm{supp}(\rho )) \gtrsim 1$ for *arbitrary* bounded self-adjoint deformations $D =D^*$ (see [21, Theorem 2.1]), the complementary regime requires the additional Assumption 2.2 on *D* stated below (see [4, Theorem 2.6] and [23, Theorem 2.8]). A sufficient condition for Assumption 2.2 is discussed in Remark 2.3; see also [3].

In the remainder of this section, we formulate our main results on the two different Loschmidt echo scenarios described in Sect. 1.

### Scenario I: Two deformations of a Wigner matrix

For the first echo scenario, we consider two deformed Wigner matrices, $H_j = D_j + W$, $j \in [2]$, and denote their resolvents and corresponding deterministic approximation (2.1) by $G_j$ and $M_j$, respectively. A natural definition of the averaged Loschmidt echo is

2.5 $$\begin{aligned} \overline{\mathfrak {M}}(t) = \overline{\mathfrak {M}}_{H_1, H_2}^{(E_0, \eta _0)}(t):= \left| \frac{\left\langle \textrm{e}^{\textrm{i}tH_1}\Im G_1(E_0 + \textrm{i}\eta _0) \textrm{e}^{-\textrm{i}tH_2}\right\rangle }{\left\langle \Im M_1(E_0+ \textrm{i}\eta _0) \right\rangle } \right| ^2\,, \end{aligned}$$

since $\Im G/\langle \Im M \rangle$ in (2.5) effectively localizes around $E_0$ and *averages* in a window of size $\eta _0 >0$ assumed to be independent of *N*. In Remark 2.7 below we comment on the averaging implemented by (2.5). Note that in order to match (1.2)-(1.3) from the introduction we need to replace *t* by $-t$ in (2.5). However, this replacement does not change the quantity (2.5) since

$$\begin{aligned} \left| \left\langle \textrm{e}^{\textrm{i}tH_1}\Im G_1(E_0 + \textrm{i}\eta _0) \textrm{e}^{-\textrm{i}tH_2}\right\rangle \right| = \left| \left\langle \textrm{e}^{\textrm{i}tH_2}\Im G_1(E_0 + \textrm{i}\eta _0) \textrm{e}^{-\textrm{i}tH_1}\right\rangle \right| =\left| \left\langle \textrm{e}^{-\textrm{i}tH_1}\Im G_1(E_0 + \textrm{i}\eta _0) \textrm{e}^{\textrm{i}tH_2}\right\rangle \right| , \end{aligned}$$

where in the last step we used that $\textrm{e}^{\textrm{i}t H_1}$ and $\Im G_1(E_0+\textrm{i}\eta _0)$ commute. Using this observation, we will work with (2.5) in the rest of the paper. The same comment applies also to the other versions of the averaged Loschmidt echo defined in Sect. 2.1, namely to (2.9a), (2.9b) and (2.12).

We will henceforth assume that the deformations $D_1, D_2$ are such that the corresponding solutions $M_1, M_2$ to (2.1) are bounded.

#### Assumption 2.2

(*Boundedness of* *M*) Let *D* be an $N\times N$ Hermitian matrix and *M* the solution to (2.1). We assume that there exists an *N*-independent positive constant *L* such that $\sup _{z\in \textbf{C}{\setminus } \textbf{R}} \Vert M(z)\Vert < L$.

Assumption 2.2 is the basis for the *shape theory* of the scDos, which we briefly described in Footnote 5. We now give a sufficient condition on *D* for Assumption 2.2 to hold. It basically requires that its ordered eigenvalue sequence has to be piecewise Hölder-1/2 continuous as a function of the label.

#### Remark 2.3

(Sufficient condition for Assumption 2.2) Denote the eigenvalues of any self-adjoint deformation *D* by $\lbrace d_j\rbrace _{j=1}^N$ labeled in increasing order, $d_j\le d_k$ for $j<k$. Fix a (large) positive constant $L>0$. The set $\mathcal {M}_L$ of admissible self-adjoint deformations *D* is defined as follows: we say that $D\in \mathcal {M}_L$ if $\Vert D\Vert \le L$ and there exists an *N*-independent partition $\lbrace I_s\rbrace _{s=1}^m$ of [0, 1] in at most *L* segments such that for any $s\in [1,m]$ and any $j,k\in [1,N]$ with $j/N,k/N\in I_s$ we have $\vert d_j-d_k\vert \le L\vert j/N- k/N\vert ^{1/2}$. Since the operator $\mathcal {S}=\langle \cdot \rangle$ is flat, condition $D\in \mathcal {M}_L$ implies that *D* satisfies Assumption 2.2 for some $L'<\infty$ by means of [3, Lemma 9.3].

We can now formulate our first main result.

#### Theorem 2.4

(Averaged Loschmidt echo with two deformations) Let *W* be a Wigner matrix satisfying Assumption 2.1, and $D_1, D_2\in \textbf{C}^{N \times N}$ be bounded, traceless^6^ Hermitian matrices, i.e., $\Vert D_j\Vert \le L$ for some $L > 0$ and $\langle D_1 \rangle = \langle D_2 \rangle = 0$, additionally satisfying Assumption 2.2. Fix $\eta _0\le 1$ and let $E_0$ be an energy in the bulk of the scDos of $H_1$ and $H_2$, i.e., assume that there exist $\delta , \kappa >0$ such that $[E_0-\delta ,E_0+\delta ] \subset \textbf{B}_\kappa (\rho _1) \cap \textbf{B}_\kappa (\rho _2)$. We also assume that parameters $\eta _0, \kappa$ and $\delta$ are *N*-independent.

Consider the deformed Wigner matrices $H_j:= D_j + W$ for $j \in [2]$ and the corresponding averaged (at energy $E_0$ in a window of size $\eta _0> 0$) Loschmidt echo $\overline{\mathfrak {M}}(t)$ for times $t \ge 0$ defined in (2.5). Then, we have the following:

(i) [Short-time parabolic decay] As $t \rightarrow 0$, it holds that
  2.6 $$\begin{aligned} \overline{\mathfrak {M}}(t) = 1 - \gamma t^2+ \mathcal {O}\big (\langle D^2 \rangle t^3\big ) + \mathcal {O}_\prec ((N \eta _0)^{-1}) \end{aligned}$$
  where the *decay parameter* is given by $\gamma := \langle \left( D - \langle P D \rangle \right) ^2 P\rangle$, where we abbreviated $D:= D_2 - D_1$ and $P:= \Im M_1(E_0 + \textrm{i}\eta _0)/\langle \Im M_1 (E_0 + \textrm{i}\eta _0) \rangle$. It satisfies $\gamma \sim \Delta ^2:= \langle D^2 \rangle$ and the implicit constant in $\sim$ depends only on $\kappa$ and *L*. The implicit constants in the error terms in (2.6) depend only on $L, \delta , \kappa$ and the $C_p$’s from Assumption 2.1.
(ii) [Intermediate-time asymptotic decay] Take a (large) positive *K* and consider times $1\le t\le K/\Delta ^2$. Then, there exists a positive constant *c* such that whenever $\Delta <c$ and $\eta _0<\Delta /\vert \log \Delta \vert$ it holds that
  2.7 $$\begin{aligned} \overline{\mathfrak {M}}(t) = \textrm{e}^{- \Gamma t} + \mathcal {O}\left( \mathcal {E}\right) + \mathcal {O}_\prec \big (C(t,\eta _0)/N\big ), \end{aligned}$$
  where the *rate* $\Gamma$ (explicitly given in (4.27)) satisfies $\Gamma \sim \Delta ^2$ with the implicit constant depending only on $\kappa$ and *L*. Moreover, we denoted
  2.8 $$\begin{aligned} \mathcal {E} = \mathcal {E}(t, \Delta , \eta _0) := \frac{1+\log t}{t}+\Delta \vert \log \Delta \vert +\frac{\eta _0\vert \log \Delta \vert }{\Delta } \end{aligned}$$
  and $C(t) > 0$ is a positive constant depending only on *t*. The implicit constants in the error terms in (2.7) depend only on $L,\delta ,\kappa ,K$ and the $C_p$’s from Assumption 2.1.

Note that Theorem 2.4 addresses only times *t* which do not depend on *N*. Reaching times of order $\log N$, i.e., accessing the saturation time would require a different proof strategy. Since $t \le K/\Delta ^2$, we find that the leading term $\textrm{e}^{-\Gamma t}$ in (2.7) remains of order one throughout the whole time regime. The error term $\mathcal {E}$ is small compared to this leading term if $t \gg 1$, $\Delta \ll 1$, and $\eta _0 \ll \Delta /|\log \Delta |$; hence, these relations define the regime of the parameters where our theorem is meaningful.

The following corollary to Theorem 2.4 reveals the key property of the Loschmidt echo, the partial recovery of the initial overlap, as discussed in the introduction; see Fig. 2.

#### Corollary 2.5

(Averaged Loschmidt echo process) Assume the set-up and the conditions of Theorem 2.4. For time $t>0$ define the *averaged Loschmidt echo process* $\overline{\mathfrak {P}}_t(s)$, $s\in [0,2t]$, as follows:

2.9a $$\begin{aligned}&\overline{\mathfrak {P}}_t(s):=\left| \frac{\left\langle \textrm{e}^{\textrm{i}sH_1}\Im G_1(E_0+\textrm{i}\eta _0)\right\rangle }{\left\langle \Im M_1(E_0+\textrm{i}\eta _0)\right\rangle }\right| ^2,\quad  &   s\in [0,t], \end{aligned}$$

2.9b $$\begin{aligned}&\overline{\mathfrak {P}}_t(s):=\left| \frac{\left\langle \textrm{e}^{\textrm{i}tH_1}\Im G_1(E_0+\textrm{i}\eta _0)\textrm{e}^{-\textrm{i}(s-t)H_2}\right\rangle }{\left\langle \Im M_1(E_0+\textrm{i}\eta _0)\right\rangle }\right| ^2,\quad  &   s\in (t,2t]. \end{aligned}$$

Let $\lim ^*$ be the simultaneous limit in $\Delta , \eta _0, t$ such that $\Delta , \eta _0\rightarrow 0$ and $t\rightarrow \infty$ under constraints $\Delta ^2\ll \eta _0\ll \Delta /\vert \log \Delta \vert$ and $1/\eta _0\ll t\lesssim 1/\Delta ^2$. Here $a\ll b$ means that $a/b\rightarrow 0$ in this limit. Then, almost surely we have

2.10 $$\begin{aligned} \textrm{lim}^*\limsup _{N\rightarrow \infty }\frac{\overline{\mathfrak {P}}_t(t)}{\overline{\mathfrak {P}}_t(2t)}=\textrm{lim}^*\limsup _{N\rightarrow \infty }\frac{\overline{\mathfrak {P}}_t(t)}{\textrm{e}^{-\Gamma t}} = 0, \end{aligned}$$

where $\Gamma$ is the same as in Theorem 2.4.

#### Proof of Corollary 2.5

Firstly take the limit $N\rightarrow \infty$ in the denominator $\overline{\mathfrak {P}}_t(2t)=\overline{\mathfrak {M}}(t)$ of (2.10). Recall the definition of $\mathcal {E}$ from (2.8). By means of Theorem 2.4 we have

$$\begin{aligned} \liminf _{N\rightarrow \infty }\overline{\mathfrak {P}}_t(2t) = \liminf _{N\rightarrow \infty }\left( \textrm{e}^{-\Gamma t}+\mathcal {O}\left( \mathcal {E}(t,\Delta ,\eta _0)\right) \right) =\liminf _{N\rightarrow \infty }\left( \textrm{e}^{-\Gamma t}(1+o(1))\right) \sim 1 \end{aligned}$$

in the limit $\lim ^*$. Here, we used that $\Gamma \sim \Delta ^2$ and $t\lesssim \Delta ^{-2}$, so $\textrm{e}^{-\Gamma t}\sim 1$. Thus, in order to verify (2.10) it is sufficient to show that

$$\begin{aligned} \textrm{lim}^*\limsup _{N\rightarrow \infty }\overline{\mathfrak {P}}_t(t)=0. \end{aligned}$$

From the average single resolvent global law for $H_1$, see (2.4) or [21, Theorem 2.1], we get that

$$\begin{aligned} \lim _{N\rightarrow \infty } \left| \left\langle \textrm{e}^{\textrm{i}tH_1}\Im G_1(E_0+\textrm{i}\eta _0)\right\rangle - \int _\textbf{R}\textrm{e}^{\textrm{i}tx}\frac{\eta _0}{(x-E_0)^2+\eta _0^2}\rho _1(x)\textrm{d}x\right| = 0. \end{aligned}$$

Recall that $E_0\in {\varvec{B}}_\kappa (\rho _1)$. Thus $\langle \Im M(E_0+\textrm{i}\eta _0)\rangle \sim 1$ for $\eta _0\rightarrow 0$ and

2.11 $$\begin{aligned} \limsup _{N\rightarrow \infty }\overline{\mathfrak {P}}_t(t)\lesssim \limsup _{N\rightarrow \infty } \left| \int _\textbf{R}\textrm{e}^{\textrm{i}tx}\frac{\eta _0}{(x-E_0)^2+\eta _0^2}\rho _1(x)\textrm{d}x\right| ^2 \lesssim \left( \frac{1}{\eta _0 t}\right) ^2. \end{aligned}$$

In the last inequality, we employed integration by parts. Additionally we used that $\rho _1(x)$ is a bounded function of *x* which is guaranteed by Assumption 2.2 and that $\rho _1(x)$ has bounded derivative for $|x-E_0| \le \delta$ (see also Footnote 5), where $\delta$ was fixed in Theorem 2.4. Both of these bounds (on $\rho _1(x)$ and $\textrm{d}\rho _1(x)/\textrm{d}x$) are uniform in *N*. In the limit $\lim ^*$ we have $\eta _0 t\rightarrow \infty$, so (2.11) finishes the proof of Corollary 2.5. $\square$

As mentioned in the introduction, we also have the following corollary to Theorem 2.4.

#### Corollary 2.6

(Scrambled averaged Loschmidt echo with two deformations) Assume the conditions of Theorem 2.4 and consider (as a variant of (2.5)) the *scrambled* averaged Loschmidt echo

2.12 $$\begin{aligned} \overline{\mathfrak {M}}_\delta ^\textrm{sc}(t) := \left| \frac{\left\langle \textrm{e}^{- \textrm{i}\delta \widetilde{W}}\textrm{e}^{\textrm{i}tH_1}\Im G_1(E_0 + \textrm{i}\eta _0) \textrm{e}^{-\textrm{i}tH_2}\right\rangle }{\left\langle \Im M_1(E_0+ \textrm{i}\eta _0) \right\rangle } \right| ^2\,, \end{aligned}$$

where $\widetilde{W}$ is a Wigner matrix satisfying Assumption 2.1, *independent* of *W* and $0\le \delta \le N^{2/3-\varepsilon }$ for some fixed $\varepsilon >0$. Moreover, let $\varphi$ be the Fourier transform of the semi-circular density of states $\rho _{\textrm{sc}}(x):= (2 \pi )^{-1} \sqrt{[4-x^2]_+}$, which is explicitly given as

2.13 $$\begin{aligned} \varphi (\delta ) := \widehat{\rho _{\textrm{sc}}}(\delta ) = \int _\textbf{R}\textrm{e}^{- \textrm{i}\delta x} \rho _{\textrm{sc}}(x) \textrm{d}x = \frac{J_1(2 \delta )}{\delta } \end{aligned}$$

where $J_1$ is the first-order Bessel function of the first kind.

Then, instead of (2.6)–(2.7), we have that

$$\begin{aligned} \overline{\mathfrak {M}}_\delta ^\textrm{sc}(t) = (\varphi (\delta ))^2 \, \big [1 - \gamma t^2 + \mathcal {O}\big (\langle D^2 \rangle t^3\big ) + \mathcal {O}_\prec ((N \eta _0)^{-1}) \big ] + \mathcal {O}_\prec \big (\delta /(N \eta _0)\big ) \quad {\text {as}} \quad t \rightarrow 0 \end{aligned}$$

and

$$\begin{aligned} \overline{\mathfrak {M}}_\delta ^\textrm{sc}(t) = (\varphi (\delta ))^2 \, \big [ \textrm{e}^{- \Gamma t} + \mathcal {O}\left( \mathcal {E}\right) + \mathcal {O}_\prec \big (C(t)/N\big )\big ] + \mathcal {O}_\prec \big (\delta /(N \eta _0)\big ) \quad {\text {for}} \quad 1 \le t \le K/\Delta ^2 \end{aligned}$$

in the short and intermediate time regimes, respectively.

#### Proof of Corollary 2.6

Denote $A:= \textrm{e}^{\textrm{i}tH_1}\Im G_1(E_0 + \textrm{i}\eta _0) \textrm{e}^{-\textrm{i}tH_2}$ and observe that $\Vert A \Vert \le 1/\eta _0$. Then, by residue calculus with the contour $C_\delta := \{ z \in \textbf{C}: \textrm{dist}(z, [-2,2]) = \delta ^{-1}\}$ and a single resolvent law^7^ as in (2.4), using only the randomness of $\widetilde{W}$, we find

$$\begin{aligned} \begin{aligned} \langle \textrm{e}^{- \textrm{i}\delta \widetilde{W}} A \rangle&= \frac{1}{2 \pi \textrm{i}} \oint _{C_\delta } \textrm{e}^{-\textrm{i}\delta z } \langle A (W-z)^{-1} \rangle \textrm{d}z \\&= \frac{\langle A \rangle }{2 \pi \textrm{i}} \oint _{C_\delta } \textrm{e}^{-\textrm{i}\delta z } m_{\textrm{sc}}(z) \textrm{d}z + \mathcal {O}_\prec (\delta /(N \eta _0)) \\&= \langle A \rangle \int _\textbf{R}\textrm{e}^{- \textrm{i}\delta x} \rho _{\textrm{sc}}(x) \textrm{d}x + \mathcal {O}_\prec (\delta /\sqrt{N}) = \langle A \rangle \varphi (\delta ) + \mathcal {O}_\prec (\delta /(N \eta _0)) \,. \end{aligned} \end{aligned}$$

The rest of the proof follows from Theorem 2.4. $\square$

We close this section by commenting on the effect of the small averaging of the Loschmidt echo over several energy states implemented in (2.5). This is a necessary technical step for our proof in Scenario I that relies on a two-resolvent global law. Note that averaging will not be necessary for Scenario II since it uses only single resolvent global law. This is because randomness is present only in the second Hamiltonian, while the first is modeled by a deterministic matrix.

#### Remark 2.7

(Averaging of the Loschmidt echo) We provide two independent non-rigorous arguments for the averaged Loschmidt echo $\overline{\mathfrak {M}}$ and the original Loschmidt echo $\mathfrak {M}$ being close to each other.

1. First, by means of the Eigenstate Thermalization Hypothesis (ETH) for a deformed Wigner matrix $H = D + W$, see [13, Theorem 2.7], and a single resolvent local law (2.4), it holds that
  2.14 $$\begin{aligned} \langle \varvec{u}_j, A \varvec{u}_j \rangle \approx \frac{\langle \Im M(E_0 + \textrm{i}\eta _0) A \rangle }{\langle \Im M(E_0 + \textrm{i}\eta _0)\rangle } \approx \frac{\langle \Im G(E_0 + \textrm{i}\eta _0) A \rangle }{\langle \Im M(E_0 + \textrm{i}\eta _0)\rangle }\,. \end{aligned}$$
  Here, *A* is an arbitrary deterministic matrix, $\varvec{u}_j$ is a (normalized) eigenvector of *H* with eigenvalue $\approx E_0$, and $\eta _0$ a small regularization. In this sense, the pure state $\mathinner {|{\varvec{u}_j}\rangle } \mathinner {\langle {\varvec{u}_j}|}$ is *weakly* close to $\Im G/\langle \Im M \rangle$ (i.e., if tested against a deterministic *A*), which heuristically supports the implementation of the averaged Loschmidt echo in (2.5). However, the rigorous ETH statements do not allow to choose *A* depending on the underlying randomness like $A=e^{-\textrm{i}t H_2} e^{\textrm{i}t H_1}$.
2. Another supporting argument uses the fact that the *averaged overlap function* $\overline{\mathfrak {S}}^{(E, \eta _0)}(t)$ (in particular its phase) is approximately constant as long as *E* varies in a range $|E-E_0| \lesssim \eta _0$. Hence, it is irrelevant if one (a) first averages and then takes absolute value square, or (b) does it the other way around. The fact that $\overline{\mathfrak {S}}^{(E, \eta _0)}(t)$ is slowly varying in *E* follows by a simple computation using that (i) $\overline{\mathfrak {S}}^{(E_0, \eta _0)}(t) \approx I_{E_0, \eta _0}(t)/\langle \Im M_1(E_0 + \textrm{i}\eta _0) \rangle$ (see (4.1) and (4.7)), (ii) $I_{E_0,\eta _0}$ is given by $\textrm{e}^{\textrm{i}t\mathfrak {s}_0} \langle \Im M_1(E_0 + \textrm{i}\eta _0) \rangle$ (see (4.26)), (iii) the exponent $\mathfrak {s}_0$ is Lipschitz continuous on scale $\Delta$ (see the last relation of (4.16)), and (iv) we have $t \lesssim \Delta ^{-2}$ and $\eta _0\ll \Delta$ by assumption.

Both, the ETH argument (2.14) and the fact that $\overline{\mathfrak {S}}^{(E, \eta _0)}(t)$ is approximately constant as long as $|E-E_0| \lesssim \eta _0$, independently indicate that the averaged Loschmidt echo $\overline{\mathfrak {M}}$ and the non-averaged Loschmidt echo $\mathfrak {M}$ should practically agree with each other. However, neither of them constitutes a rigorous proof, since (1) the observable *A* in (2.14) cannot be chosen to depend on the randomness, and (2) we cannot exclude that for some initial fixed energy state $\psi _0$, $\mathfrak {S}$ in (1.2) behaves very differently from its typical value computed by local averaging.

### Scenario II: Perturbation by a Wigner matrix

For the second echo scenario, we consider a single deformed Wigner matrix $H_\lambda = H_0 + \lambda W$ and the Loschmidt echo

2.15 $$\begin{aligned} \mathfrak {M}(t) = \mathfrak {M}_{H_\lambda , H_0}^{(E_0, \Delta )}(t) := \left| \langle \psi _0, \textrm{e}^{\textrm{i}t H_\lambda } \textrm{e}^{- \textrm{i}t H_0} \psi _0 \rangle \right| ^2 \end{aligned}$$

for some normalized initial state $\psi _0 \in \textbf{C}^N$ with energy $E_0 = \langle \psi _0, H_0 \psi _0 \rangle$ and localized in an interval of size $\Delta$ around $E_0$ (see Assumption 2.9 below for a precise statement). The localization parameter $\Delta$ plays the same role as $\eta _0$ in Sect. 2.1, but here we work with a sharp cutoff in the energy.

The unperturbed Hamiltonian $H_0$ is assumed to satisfy the following.

#### Assumption 2.8

($$H_0$$ *and its limiting density of states*) The Hamiltonian $$H_0 $$ is deterministic, self-adjoint $$H_0 = H_0^*$$, and uniformly bounded, $$\Vert H_0 \Vert \le C_{H_0}$$ for some $$C_{H_0} > 0$$. We denote the resolvent of $$H_0$$ at any spectral parameter $$z \in \textbf{C}\setminus \textbf{R}$$ by $$M_0(z):= (H_0 -z)^{-1}$$. Moreover, we assume the following:

(i) There exists a compactly supported measurable function $\rho _0: \textbf{R}\rightarrow [0,+\infty )$ with $\int _\textbf{R}\rho _0(x) \textrm{d}x = 1$ and two positive sequences $\epsilon _0(N)$ and $\eta _0(N)$, both converging to zero as $N\rightarrow \infty$, such that, uniformly in $z \in \textbf{C}\backslash \textbf{R}$ with $\eta :=|\Im z| \ge \eta _0 \equiv \eta _0(N)$, we have
  2.16 $$\begin{aligned} \langle M_0(z)\rangle = m_0(z) + \mathcal {O}(\epsilon _0) \quad {\text {with}} \quad \epsilon _0 \equiv \epsilon _0(N)\,. \end{aligned}$$
  Here,
  2.17 $$\begin{aligned} m_0(z):= \int _\textbf{R}\frac{\rho _0(x)}{x-z}\textrm{d}x \end{aligned}$$
  is the Stieltjes transform of $\rho _0$. We refer to $\rho _0$ as the *limiting density of states*, and to $\textrm{supp}(\rho _0)$ as the *limiting spectrum* of $H_0$.
(ii) For small positive constants $\kappa ,c>0$, we define the set of *admissible energies* $\sigma _{\textrm{adm}}^{(\kappa ,c)}$ in the limiting spectrum of $H_0$ by^8^
  2.18 $$\begin{aligned} \sigma _{\textrm{adm}}^{(\kappa ,c)} := \left\{ x \in \textrm{supp}(\rho _0) : \inf _{|y-x|\le \kappa }\rho _0(y) > c,\, \Vert \rho _0\Vert _{C^{1,1}([x-\kappa , x+\kappa ])} \le 1/c\right\} . \end{aligned}$$
  We assume that for some positive *N*-independent $\kappa ,c> 0$, $\sigma _{\textrm{adm}}^{(\kappa ,c)}$ is not empty.

Assuming that the set of admissible energies in (2.18) is non-empty guarantees the limiting spectrum $\textrm{supp} (\rho _0)$ has a part, where the limiting density of states behaves regularly, i.e., it is sufficiently smooth and strictly positive (in the *bulk*).

#### Assumption 2.9

(*Locality of the initial state*) Given Assumption 2.8, we first pick a *reference energy*

2.19 $$\begin{aligned} E_0 \in \sigma _{\textrm{adm}}^{(\kappa _0,c_0)} \quad {\text {for some}} \quad \kappa _0, c_0>0, \end{aligned}$$

and further introduce $I_\delta :=[E_0-\delta ,E_0+\delta ]$ for any $0<\delta <\kappa _0$. Moreover, take an *N*-independent *energy width* $\Delta \in (0, \kappa _0/2)$ and let $\Pi _\Delta := \textbf{1}_{I_\Delta }(H_0)$ be the spectral projection of $H_0$ onto the interval $I_\Delta$. Then, we assume that the initial state $\psi _0 \in \textbf{C}^{N}$ is normalized, $\Vert \psi _0 \Vert = 1$, has energy $E_0 = \langle \psi _0, H_0 \psi _0 \rangle$, and satisfies $\Pi _\Delta \psi _0 = \psi _0$, i.e., $\psi _0$ is localized in $I_\Delta$.

#### Theorem 2.10

(Loschmidt echo with a single deformation) Consider the Loschmidt echo (2.15) for times $t \ge 0$ and assume that its constituents satisfy Assumptions 2.1 and 2.8–2.9. Then, we have the following:

(i) [Short-time parabolic decay] As $t \rightarrow 0$ it holds that
  2.20 $$\begin{aligned} \mathfrak {M}(t) = 1 - \lambda ^2 t^2 + \mathcal {O}(\lambda ^2 t^3) + \mathcal {O}_\prec \big ( 1/\sqrt{N}\big ) \,. \end{aligned}$$
  The implicit constants in the error terms in (2.20) only depend on $C_{H_0}$ and the $C_p$’s from Assumption 2.1.
(ii) [Intermediate-time asymptotic decay] For all times $t \ge 0$, it holds that
  2.21 $$\begin{aligned} \mathfrak {M}(t) = \textrm{e}^{-2\pi \rho _0(E_0) \lambda ^2 t} + \mathcal {O}(\mathcal {E}) + \mathcal {O}_\prec \big (C(t, \lambda )/\sqrt{N}\big ) \,, \end{aligned}$$
  where for any fixed $T > 0$ the error term $\mathcal {E}$, explicitly given in (7.8), satisfies
  $$\begin{aligned} \lim \limits _{\Delta \rightarrow 0} \lim \limits _{\begin{array}{c} t \rightarrow \infty , \lambda \rightarrow 0 \\ \lambda ^2 t \le T \end{array}} \lim \limits _{N \rightarrow \infty } \mathcal {E} = 0 \end{aligned}$$
  and the constant $C(t, \lambda )> 0$ depends only on its arguments. The implicit constants in the error terms in (2.21) depend only on $C_{H_0}$ from Assumption 2.8, $\kappa _0, c_0$ from Assumption 2.9, and the $C_p$’s from Assumption 2.1.

In the small time regime, $t \rightarrow 0$, (2.20) is surely more precise than (2.21), but the latter is more relevant to describe the exponential decay for times of order $t \sim \lambda ^{-2}$.

The proof of Corollary 2.11 is completely analogous to the proof of Corollary 2.6 (only using an isotropic law instead of an averaged law) and so omitted.

#### Corollary 2.11

(Scrambled Loschmidt echo with a single deformation) Assume the conditions of Theorem 2.10 and consider (as a variant of (2.15)) the *scrambled* Loschmidt echo

2.22 $$\begin{aligned} \mathfrak {M}_\delta ^\textrm{sc}(t) := \left| \left\langle \psi _0, \textrm{e}^{\textrm{i}t H_\lambda } \textrm{e}^{-\textrm{i}\delta \widetilde{W}}\textrm{e}^{- \textrm{i}t H_0} \psi _0 \right\rangle \right| ^2\,, \end{aligned}$$

where $\widetilde{W}$ is a Wigner matrix satisfying Assumption 2.1, *independent* of *W* and $0\le \delta \le N^{2/3-\varepsilon }$ for some fixed $\varepsilon >0$. Moreover, let $\varphi$ be given by (2.13).

Then, instead of (2.20)–(2.21), we have that

$$\begin{aligned} \mathfrak {M}_\delta ^\textrm{sc}(t) = (\varphi (\delta ))^2 \, \big [1 - \lambda ^2 t^2 + \mathcal {O}(\lambda ^2 t^3) + \mathcal {O}_\prec \big ( 1/\sqrt{N}\big ) \big ] + \mathcal {O}_\prec \big (\delta /\sqrt{N}\big ) \quad {\text {as}} \quad t \rightarrow 0 \end{aligned}$$

and

$$\begin{aligned} \mathfrak {M}_\delta ^\textrm{sc}(t) = (\varphi (\delta ))^2 \, \big [ \textrm{e}^{-2\pi \rho _0(E_0) \lambda ^2 t} + \mathcal {O}(\mathcal {E}) + \mathcal {O}_\prec \big (C(t, \lambda )/\sqrt{N}\big )\big ] + \mathcal {O}_\prec \big (\delta /\sqrt{N}\big ) \quad {\text {for}} \quad \lambda ^2 t \le T, \end{aligned}$$

respectively.

The rest of the paper is devoted to proving Theorems 2.4 and 2.10. The proof of Theorem 2.4 is conducted in Sects. 3–4. In Sect. 7, we prove Theorem 2.10. The proof of several technical results from Sect. 4 is deferred to Sects. 5 and 6, and Appendix A.

## Short-time parabolic decay in Scenario I: Proof of Theorem 2.4 (i)

In the following, we abbreviate $\widetilde{P} = \Im G_1(E_0 + \textrm{i}\eta _0)/\langle \Im M_1(E_0 + \textrm{i}\eta _0) \rangle$, such that $\mathfrak {M}(t)$ can be written as

3.1 $$\begin{aligned} \overline{\mathfrak {M}}(t) = \left| \left\langle \textrm{e}^{\textrm{i}tH_1} \widetilde{P} \textrm{e}^{-\textrm{i}tH_2}\right\rangle \right| ^2 = \left| \left\langle \widetilde{P}\textrm{e}^{\textrm{i}tH_1} \textrm{e}^{-\textrm{i}tH_2}\right\rangle \right| ^2 \,. \end{aligned}$$

Next, we trivially Taylor expand $\textrm{e}^{ \textrm{i}tH_1}$ and $\textrm{e}^{-\textrm{i}t H_2}$ to second order, leaving us with

3.2 $$\begin{aligned} \textrm{e}^{ \textrm{i}tH_1} \textrm{e}^{-\textrm{i}t H_2} = 1 + \textrm{i}t(H_1 - H_2) - \frac{t^2}{2} \big ( (H_1 -H_2)^2 - [H_1, H_2] \big ) + \mathcal {O}(t^3) \,. \end{aligned}$$

Plugging this in (3.1), we find

3.3 $$\begin{aligned} \begin{aligned} \overline{\mathfrak {M}}(t) =&\left\langle \widetilde{P} \left( 1 - \tfrac{t^2}{2}((H_1 - H_2)^2 - [H_1, H_2])\right) \right\rangle ^2 \\&\qquad + t^2 \langle \widetilde{P} (H_1 -H_2) \rangle ^2 + \mathcal {O}(t^3) + \mathcal {O}_\prec ((N \eta _0)^{-1}) \\ =&1 - \big \langle \left( D - \langle P D \rangle \right) ^2 P \big \rangle \, t^2 + \mathcal {O}(t^3) + \mathcal {O}_\prec ((N \eta _0)^{-1}) \,. \end{aligned} \end{aligned}$$

Here, we additionally used that $\langle \widetilde{P} [H_1,H_2] \rangle = 0$ since $\widetilde{P}$ is a function of $H_1$, $D= H_2 - H_1$, and a single resolvent law in the form $\langle \widetilde{P} A \rangle = \langle P A \rangle + \mathcal {O}_\prec ((N \eta _0)^{-1})$ for any *A* with $\Vert A \Vert \lesssim 1$. The fact that the decay parameter $\gamma = \langle \left( D - \langle P D \rangle \right) ^2 P\rangle$ satisfies $\gamma \sim \Delta ^2$ is a simple consequence of the *flatness* of the stability operator for a deformed Wigner matrix (see, e.g., [3, Proposition 3.5]).

In order to conclude (2.6), it remains to show that the error term $\mathcal {O}(t^3)$ in (3.3) is actually improvable to $\mathcal {O}(\langle D^2\rangle t^3)$. To see this, we (formally)^9^ employ the Baker–Campbell–Hausdorff (BCH) formula, to write the exponentials as

3.4 $$\begin{aligned} \begin{aligned} \textrm{e}^{ \textrm{i}tH_1} \textrm{e}^{-\textrm{i}t H_2} =&\, \textrm{e}^{K} \quad {\text {with}} \\ K =&\, \textrm{i}t (H_1 - H_2) + \frac{t^2}{2} [H_1 , H_2] + \frac{\textrm{i}t^3}{12} \big ( [H_1, [H_1, H_2]] - [H_2, [H_1, H_2]]\big ) \\&- \frac{t^4}{24} [H_2, [H_1, [H_1, H_2]]] + ... \end{aligned} \end{aligned}$$

and note that every summand in the expression for *K* in (3.4) can be written as a linear combination of nested commutators of *D* with $H \equiv H_1$ with one *D* always being in the innermost commutator. Hence, to conclude the desired, we need to show that (i) all the terms in $\textrm{e}^K$ containing only a single *D* vanish, when evaluated in $\langle \widetilde{P}... \rangle$, and (ii) all the terms in $\textrm{e}^K$ containing at least two *D*’s lead to an additional $\langle D^2\rangle$-factor in the error term.

For (i), note that the only way to have just a single *D* in a nested commutator is precisely $\textrm{ad}_H^n(D)$ with $\textrm{ad}_H(D):= [H,D]$. Evaluated in $\langle \widetilde{P}... \rangle$, this vanishes, $\langle \widetilde{P} \textrm{ad}_H^n(D) \rangle = 0$, since $[\widetilde{P}, H] = 0$ and hence

3.5 $$\begin{aligned} \langle \widetilde{P} \textrm{ad}_H^n(D) \rangle = \sum _{k=0}^n \left( {\begin{array}{c}n\\ k\end{array}}\right) (-1)^k \langle \widetilde{P} H^{n-k} D H^k \rangle = \langle \widetilde{P} H^n D \rangle \sum _{k=0}^n \left( {\begin{array}{c}n\\ k\end{array}}\right) (-1)^k = 0\,. \end{aligned}$$

For (ii), we take a product, say, *T*, of *H*’s and at least two *D*’s, resulting from resolving (a product of) nested commutators, and estimate

3.6 $$\begin{aligned} \begin{aligned} \left| \langle \widetilde{P} T \rangle \right| \lesssim \langle \widetilde{P} D^2 \rangle = \langle P D^2 \rangle + \mathcal {O}_\prec \big ((N \eta _0)^{-1}\big ) \lesssim \langle D^2 \rangle + \mathcal {O}_\prec \big ((N \eta _0)^{-1}\big )\,. \end{aligned} \end{aligned}$$

In the first step, we estimated all *H*’s and all but two *D*’s in *T* by their operator norm, additionally using that $\widetilde{P} \ge 0$ and $[H, \widetilde{P}] = 0$. In the second step, we employed the single resolvent law (2.4), while in the last step we used $\Vert P\Vert \lesssim 1$.

We have hence shown that all the terms of $\textrm{e}^K$ in (3.4) carrying at least a third power of *t*, can in fact be bounded with an additional $\langle D^2 \rangle$-factor compared to (3.3). This concludes the proof. $\square$

## Asymptotic decay in Scenario I: Proof of Theorem 2.4 (ii)

The principal goal of this section is to prove (2.7) in Theorem 2.4 (ii), i.e., study the behavior of $\overline{\mathfrak {M}}(t)$ defined in (2.5) for times $1\le t\lesssim \Delta ^{-2}$. In order to do so, we compute the random quantity $\left\langle \textrm{e}^{\textrm{i}tH_1}\Im G_1(E_0+\textrm{i}\eta _0)\textrm{e}^{- \textrm{i}tH_2}\right\rangle$ by residue calculus as

4.1 $$\begin{aligned} \begin{aligned}&\left\langle \textrm{e}^{\textrm{i}tH_1}\Im G_1(E_0+\textrm{i}\eta _0)\textrm{e}^{- \textrm{i}tH_2}\right\rangle \\&\quad = \left( \frac{1}{2\pi \textrm{i}}\right) ^2 \!\!\!\oint _{\gamma _1}\oint _{\gamma _2}\!\!\textrm{e}^{\textrm{i}t(z_1-z_2)}\frac{\eta _0}{(z_1-E_0)^2 +\eta _0^2}\left\langle G_1(z_1)G_2(z_2)\right\rangle \textrm{d}z_1\textrm{d}z_2\\&\qquad + \frac{1}{4\pi } \oint _{\gamma _2} \textrm{e}^{\textrm{i}t(E_0+i\eta _0-z_2)}\left\langle G_1(E_0+\textrm{i}\eta _0)G_2(z_2)\right\rangle \textrm{d}z_2. \end{aligned} \end{aligned}$$

**Fig. 3:**
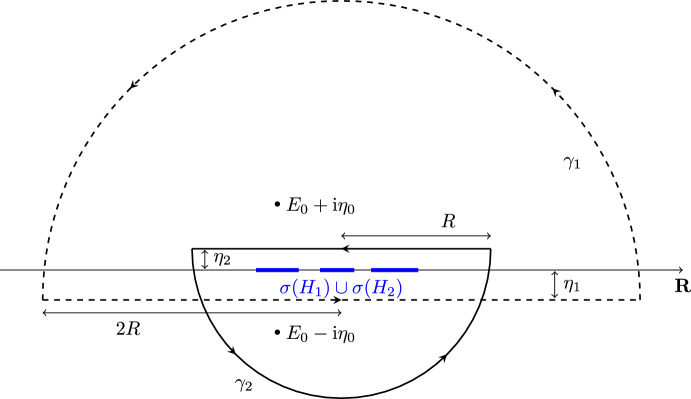
Sketch of the contours $\gamma _1$ (dashed) and $\gamma _2$ (full) from (4.2)–(4.3). The union of the spectra of $H_1$ and $H_2$ is indicated in blue

Here, the contours $\gamma _1, \gamma _2$ are chosen to be two semicircles as indicated in Fig. 3. More precisely, we take a (large) constant $R>0$ such that $\textrm{supp}\rho _1$ and $\textrm{supp}\rho _2$ are contained in $[-(R-1),R-1]$. The distance of the flat pieces from the real axis are denoted by $\eta _1:=\min \lbrace 1/t, \eta _0/2\rbrace$ and $0<\eta _2\lesssim 1/t$. The latter will explicitly be chosen later in Sect. 4.3, where we conclude the proof of Theorem 2.4 (ii). We decompose both contours into their flat in semicircular parts, $\gamma _j = \gamma _j^{(1)} \dot{+} \gamma _j^{(2)}$, $j \in [2]$, and parametrize them as follows:

4.2 $$\begin{aligned} \gamma _1^{(1)}&: \ z_1=E_1-\textrm{i}\eta _1 \quad  &   {\text {with}} \quad E_1\in [-2R,2R]\,, \quad  &   \gamma _1^{(2)} : \ z_1=2R\textrm{e}^{\textrm{i}\varphi }-\textrm{i}\eta _1 \quad  &   {\text {with}} \quad \varphi \in [0,\pi ] \end{aligned}$$

4.3 $$\begin{aligned} \gamma _2^{(1)}&: \ z_2=E_2+\textrm{i}\eta _2 \quad  &   {\text {with}} \quad E_2\in [-R,R]\,, \quad  &   \gamma _2^{(2)} : \ z_2=R\textrm{e}^{\textrm{i}\varphi }+\textrm{i}\eta _2 \quad  &   {\text {with}} \quad \varphi \in [0,\pi ] \end{aligned}$$

Finally, we point out that, in order to (4.1) being valid, $\gamma _1$ is chosen in such a way that it encircles $E_0+\textrm{i}\eta _0$, but *not* $E_0-\textrm{i}\eta _0$.

The following argument leading toward the proof of Theorem 2.4 (ii) is split in three parts. First, in Sect. 4.1, we approximate the random contour integrals (4.1) by their deterministic counterparts by using an appropriate *two resolvent global law* for two different deformations (Proposition 4.1). Afterward, in Sect. 4.2, we collect some preliminary stability bounds (Proposition 4.2) and information on the *shift*, which is the key parameter in our analysis of the Loschmidt echo; see Lemmas 4.4–4.6. Finally, in Sect. 4.3, we summarize the evaluation of the deterministic contour integrals from Sect. 4.1 in five Lemmas 4.7–4.11. Combining these with estimates on the shift from Sect. 4.2, we conclude the proof of Theorem 2.4 (ii) at the end of Sect. 4.3.

### Step (i): Global law with two deformations

The following two resolvent global law will be used to approximate (4.1) by its deterministic counterpart.

#### Proposition 4.1

(Average two resolvent global law) Let $D_1, D_2\in \textbf{C}^{N \times N}$ be a bounded Hermitian matrices, i.e., $\Vert D_j\Vert \le L$ for some $L > 0$, and *W* a Wigner matrix satisfying Assumption 2.1. Moreover, let $z_1, z_2 \in \textbf{C}$ be spectral parameters satisfying $\kappa := \min _{i \in [2]} \textrm{dist}(z_i, [-(L+2), L+2]) \ge \delta > 0$ and denote $G_j(z_j):= (D_j + W - z_j)^{-1}$ for $j \in [2]$. Then, it holds that

4.4 $$\begin{aligned} \left| \langle G_1(z_1) G_2(z_2) \rangle - \langle M(z_1, z_2)\rangle \right| \prec \frac{C(\delta )}{N}\,, \end{aligned}$$

where $C(\delta )> 0$ is a constant depending^10^ only on its argument (apart from *L* and the constants from Assumption 2.1). In (4.4), we abbreviated

4.5 $$\begin{aligned} M(z_1, z_2) = M_{12}(z_1, z_2):= \frac{M_1(z_1) M_2(z_2)}{1 - \langle M_1(z_1) M_2(z_2) \rangle } \end{aligned}$$

and $M_j = M_j(z_j)$, for $j \in [2]$, is the unique solution to the Matrix Dyson equation (MDE)

4.6 $$\begin{aligned} - \frac{1}{M_j} = z_j - D_j + \langle M_j \rangle \quad {\text {with}} \quad \Im M_j(z_j) \Im z_j > 0 \quad {\text {for}} \quad z_j \in \textbf{C}\setminus \textbf{R}\,. \end{aligned}$$

#### Proof

Using that $\Vert D_j + W \Vert \le L +2+ \epsilon$, $j \in [2]$ with very high probability and the stability bound $|1 - \langle M_1(z_1) M_2(z_2)\rangle |^{-1} \lesssim 1$ for $\kappa := \min _{i \in [2]} \textrm{dist}(z_i, [-(L+2), L+2]) \gtrsim 1$ from Proposition 4.2 below,^11^ the proof works in the same way as [22, Proposition 3.1], [12, Appendix B], [14, Section 5.2], or [13, Section 6.2]. We omit the details for brevity. $\square$

Hence, by means of Proposition 4.1, we find that the random contour integral (4.1) can be approximated by the deterministic quantity

4.7 $$\begin{aligned} \begin{aligned} I_{E_0,\eta _0}(t):=&\left( \frac{1}{2\pi \textrm{i}}\right) ^2 \oint _{\gamma _1}\oint _{\gamma _2}\textrm{e}^{\textrm{i}t(z_1-z_2)}\frac{\eta _0}{(z_1-E_0)^2 +\eta _0^2}\left\langle M(z_1,z_2)\right\rangle \textrm{d}z_1\textrm{d}z_2\\&\quad + \frac{1}{4\pi } \oint _{\gamma _2} \textrm{e}^{\textrm{i}t(E_0+i\eta _0-z_2)}\left\langle M(E_0+\textrm{i}\eta _0,z_2)\right\rangle \textrm{d}z_2. \end{aligned} \end{aligned}$$

up to an error of size $\mathcal {O}_\prec \big (C(\eta _1) C(\eta _2)/N\big )$, where we additionally used that the lengths of the contours are bounded, $\ell (\gamma _j) \lesssim 1$ for $j \in [2]$.

### Step (ii): Preliminary bounds on the stability operator and the shift

As usual in random matrix theory, local/global laws are governed by a *stability operator*, which, in our case is given by

4.8 $$\begin{aligned} \mathcal {B}_{12}(z_1, z_2)[\cdot ] := \textbf{1} - M_1 \langle \cdot \rangle M_2 \quad {\text {with}}\quad M_j \equiv M_j(z_j)\,. \end{aligned}$$

One can easily see that $\mathcal {B}_{12}(z_1, z_2)$ has a highly degenerate eigenvalue one, and its only non-trivial eigenvalue is given by $1 - \langle M_1 M_2\rangle$ with corresponding eigen“vector" $M_1 M_2$.

The following proposition, whose proof is given in Sect. 5, states an upper bound on the inverse of this non-trivial eigenvalue. A simplified form this stability bound already appeared in [11, Lemma 5.2] for the very special case that $D_1 = \alpha D_2$ for some $\alpha \in \textbf{R}$.

#### Proposition 4.2

(Stability bound) Fix a (large) $L>0$. Uniformly in $z_1,z_2\in \textbf{C}\setminus \textbf{R}$ and traceless Hermitian $D_1,D_2$ with $\vert z_j\vert \le L$, $\Vert D_j\Vert \le L$, $j=1,2$, it holds that

4.9 $$\begin{aligned} \left| \frac{1}{1-\langle M_1M_2\rangle }\right| \lesssim \frac{1}{\Delta ^2 + \left( \Re z_1-\Re z_2\right) ^2 + \left( \Im \langle M_1\rangle + \Im \langle M_2\rangle \right) ^2+\left| \frac{ \Im z_1}{\langle \Im M_1\rangle }\right| + \left| \frac{ \Im z_2}{\langle \Im M_2\rangle }\right| }\vee 1\,, \end{aligned}$$

where we denoted $\Delta ^2:=\langle (D_1-D_2)^2\rangle$.

In the current Sect. 4, more precisely, the proof of Proposition 4.1 above, only the special case

4.10 $$\begin{aligned} \left| 1-\langle M_1M_2\rangle \right| ^{-1} \lesssim 1 \quad {\text {for}} \quad \max _{j \in [2]} \textrm{dist}(z_j, \textrm{supp}(\rho _j)) \gtrsim 1 \end{aligned}$$

of Proposition 4.2 is relevant. However, for later reference, we also point out that, in particular, $\vert 1-\langle M_1M_2\rangle \vert ^{-1}\lesssim \vert z_1-z_2\vert ^{-2}$ and that the lhs. of (4.9) is bounded by one, whenever $z_1,z_2$ are in the same half-plane and $\rho _1(z_1) + \rho _2(z_2) \gtrsim 1$ (e.g., if one of them is in the bulk, $\Re z_j \in \textbf{B}_\kappa (\rho _j)$).

In addition to these bounds, Proposition 4.2 also plays an important role in the analysis of the *shift* $\mathfrak {s}(z_1,z_2)$ of the spectral parameters $z_1, z_2$ in the *(generalized)* *M*-*resolvent identity*

4.11 $$\begin{aligned} \langle M_{12}\rangle = \frac{\langle M_1M_2\rangle }{1-\langle M_1M_2\rangle } = \frac{\langle M_1\rangle - \langle M_2\rangle }{z_1-z_2-\mathfrak {s}(z_1,z_2)}, \end{aligned}$$

which can easily be obtained by subtracting MDEs (4.6) for $M_2$ and $M_1$ from each other. In (4.11), the shift is defined as follows.

#### Definition 4.3

(The *shift*) Let $D_1, D_2$ be Hermitian traceless matrices and let $M_j(z_j)$ for $j \in [2]$ be the solution of the MDE (4.6). Then, we define the *shift* (depending on $D_1, D_2$ and $z_1, z_2 \in \textbf{C}{\setminus } \textbf{R}$) as

4.12 $$\begin{aligned} \mathfrak {s}(z_1,z_2):=\frac{\langle M_1(z_1)(D_1-D_2)M_2(z_2)\rangle }{\langle M_1(z_1)M_2(z_2)\rangle }\,, \end{aligned}$$

whenever the denominator does not vanish.

As already mentioned above, the shift $\mathfrak {s}$ is the key parameter in our analysis of the Loschmidt echo. We now collect several estimates on $\mathfrak {s}$ in Lemmas 4.4–4.6. The proofs, which are based on the stability bound in Proposition 4.2, are given in Sect. 5.

#### Lemma 4.4

(Properties of $\mathfrak {s}(z_1,z_2)$) Fix a (small) $\kappa >0$ and a (large) $L>0$. Consider spectral parameters $z_1,z_2 \in \textbf{C}{\setminus } \textbf{R}$ such that $\Im z_1 \Im z_2<0$ and $\vert z_j\vert \le L$, $\Vert D_j\Vert \le L$, for $j \in [2]$. Assume that at least one of these parameters is such that the (positive) harmonic extension of the scDos is positive, i.e., $\rho _1(z_1)+\rho _2(z_2)\ge \kappa$. Then, there exists a positive constant $\mathfrak {c}$ which depends only on $\kappa , L$ such that for any Hermitian traceless $D_1,D_2$ with $\Delta := \langle (D_1 -D_2)^2 \rangle ^{1/2}\le \mathfrak {c}$ we have the following:

1. The denominator of the shift (4.12) is of order one, $\vert \langle M_1(z_1)M_2(z_2)\rangle \vert \sim 1$. In particular,
  4.13 $$\begin{aligned} \vert \mathfrak {s}(z_1,z_2)\vert \lesssim \Delta . \end{aligned}$$
2. If $$\rho _j(z_j) \ge \kappa /2$$, then
  4.14 $$\begin{aligned} \vert \partial _{z_j} \mathfrak {s}(z_1,z_2)\vert \lesssim \Delta . \end{aligned}$$

Here all implicit constants depend only on $\kappa$ and *L*.

We now introduce an auxiliary function *f*, which exactly detects the influence of the shift on the real part of a spectral parameter.

#### Lemma 4.5

(Definition of *f* and $\mathfrak {s}_0$) Fix a (small) $\kappa >0$ and a (large) $L>0$. Consider $0< \eta _1, \eta _2<L$ and a spectral parameter $z_2=E_2+\textrm{i}\eta _2$ such that $\rho _2(z_2)\ge \kappa$, and satisfying $\vert z_2\vert \le L$. Let $D_1, D_2$ be Hermitian traceless matrices with $\Vert D_j\Vert \le L$, $j\in [2]$. Assume that $\Delta := \langle (D_1 -D_2)^2 \rangle ^{1/2} \le \mathfrak {c}$, where $\mathfrak {c}$ is the constant from Lemma 4.4.

Then, there exists a unique *energy renormalization* $f^{\eta _1,\eta _2}(E_2)= f(E_2)\in \mathbb {R}$ with $\vert f(E_2)\vert \le L$ such that

$$\begin{aligned} \Re \left( f(E_2)-E_2-\mathfrak {s}(f(E_2)-\textrm{i}\eta _1,E_2+\textrm{i}\eta _2))\right) =0. \end{aligned}$$

Moreover, denoting the *renormalized (one point) shift* by

4.15 $$\begin{aligned} \mathfrak {s}_0^{\eta _1,\eta _2}(E_2):=\mathfrak {s}(f(E_2)-\textrm{i}\eta _1,E_2+\textrm{i}\eta _2), \end{aligned}$$

the functions $f^{\eta _1,\eta _2}(E_2)$ and $\mathfrak {s}_0^{\eta _1,\eta _2}(E_2)$ are differentiable in $\eta _1,\eta _2$ and for $E_2 \in \textbf{B}_\kappa (\rho _2)$ in the bulk, and the derivatives satisfy

4.16 $$\begin{aligned} \vert \partial _{E_2}f^{\eta _1,\eta _2}(E_2) - 1\vert \lesssim \Delta ,\quad \left| \partial _{\eta _j} f^{\eta _1,\eta _2}(E_2)\right| \lesssim \Delta ,\,j\in [2],\quad {\text {and}} \quad \vert \partial _{E_2}\mathfrak {s}_0^{\eta _1,\eta _2}(E_2)\vert \lesssim \Delta . \end{aligned}$$

Whenever it does not lead to confusion our ambiguities, we will omit the superscripts $\eta _1,\eta _2$ of $f^{\eta _1,\eta _2}$ and $\mathfrak {s}_0^{\eta _1,\eta _2}$. Next, we show that the *imaginary part* of the renormalized shift is in fact much smaller than indicated by the upper bounds of order $\Delta$ in (4.13)–(4.14) and (4.16).

#### Lemma 4.6

(Behavior of $\Im \mathfrak {s}_0$) Fix a (small) $\kappa >0$ and a (large) $L>0$. Let $E \in \textbf{B}_\kappa (\rho _2)$ be in the bulk of $\rho _2$. Then, there exist positive constants $c_1, c_2 > 0$ such that for any Hermitian traceless $D_1, D_2$ with $\Vert D_j\Vert \le L$, $j=1,2$, $\Delta <c_1$ and for any $0<\eta _j\le c_2\Delta$, for $j \in [2]$, it holds that

4.17 $$\begin{aligned} \Im \mathfrak {s}_0^{\eta _1,\eta _2}(E)\sim \Delta ^2. \end{aligned}$$

Here, $c_1,c_2$ and the implicit constants in (4.17) depend only on $\kappa$ and *L*.

In the following section, armed with the preliminary bounds from Proposition 4.2 and Lemmas 4.4–4.6, we carry out the evaluation of the contour integrals in (4.7).

### Step (iii): Contour integration of the deterministic approximation

Throughout this section, let [*a*, *b*] be an interval with length of order one satisfying $\textrm{dist}(E_0, [a,b]^c) \gtrsim 1$ and $\textrm{dist}([a,b], (\textrm{supp}(\rho _1) \cap \textrm{supp}(\rho _2))^c) \gtrsim 1$. That is, the energy $E_0$ from Theorem 2.4 is order one away from the boundary of [*a*, *b*] and [*a*, *b*] is simultaneously in the bulk of $\rho _1$ and $\rho _2$. The existence of such an interval is always guaranteed.

As already mentioned above, we now dissect the evaluation of (4.7) in several parts. As the first step, we show that the second line of (4.7) is in fact negligible. The proofs of Lemma 4.7 and all the other Lemmas 4.8–4.11 are given in Sect. 6.

#### Lemma 4.7

(The second line is negligible) Under the assumptions of Theorem 2.4 (ii) it holds that

$$\begin{aligned} I_{E_0}^{(2)}:= \frac{1}{4\pi } \oint _{\gamma _2} \textrm{e}^{\textrm{i}t(E_0+\textrm{i}\eta _0-z_2)}\left\langle M(E_0+i\eta _0,z_2)\right\rangle \textrm{d}z_2 = \mathcal {O}\left( \frac{1}{t}\right) . \end{aligned}$$

For the remaining first line of (4.7), we then find that the main contribution of the $\gamma _2$ integral comes from the interval $[a,b] + \textrm{i}\eta _2$, i.e., we can cut away the tails.

#### Lemma 4.8

(Cutting tails) Under the assumptions of Theorem 2.4 (ii) it holds that

$$\begin{aligned} \begin{aligned} I_{E_0}^{(1)}:=&\left( \frac{1}{2\pi \textrm{i}}\right) ^2 \oint _{\gamma _1}\oint _{\gamma _2}\textrm{e}^{\textrm{i}t(z_1-z_2)}\frac{\eta _0}{(z_1-E_0)^2 +\eta _0^2}\langle M(z_1,z_2)\rangle \textrm{d}z_1\textrm{d}z_2\\ =&\left( \frac{1}{2\pi \textrm{i}}\right) ^2 \oint _{\gamma _1}\int _a^b \textrm{e}^{\textrm{i}t(z_1-E_2-i\eta _2)}\frac{\eta _0}{(z_1-E_0)^2 +\eta _0^2}\langle M(z_1,E_2+\textrm{i}\eta _2)\rangle \textrm{d}z_1\textrm{d}E_2\\&+ \mathcal {O}\left( \frac{1}{t}+\frac{\eta _0}{\Delta }\right) . \end{aligned} \end{aligned}$$

The following lemma formally implements inside the integral from Lemma 4.8 the approximation

$$\begin{aligned} \langle M(z_1, E_2 + \textrm{i}\eta _2)\rangle = \frac{\langle M_1(z_1)\rangle - \langle M_2(E_2 + \textrm{i}\eta _2)\rangle }{z_1-(E_2 + \textrm{i}\eta _2)-\mathfrak {s}(z_1,E_2 + \textrm{i}\eta _2)} \approx \frac{\langle M_1(z_1)\rangle - \langle M_2(E_2+\textrm{i}\eta _2)\rangle }{z_1-(E_2+\textrm{i}\eta _2)-\mathfrak {s}_0^{\eta _1,\eta _2}(E_2)}\,, \end{aligned}$$

which is valid in the main contributing regime $E_1\approx E_2$. This is our *first replacement* $\mathfrak {s}(z_1,E_2 + \textrm{i}\eta _2) \rightarrow \mathfrak {s}_0^{\eta _1,\eta _2}(E_2)$.

#### Lemma 4.9

(First replacement) Denote $\mathfrak {d}:=\min _{E_2\in [a,b]}\vert \eta _1+\eta _2+\Im \mathfrak {s}_0^{\eta _1, \eta _2}(E_2)\vert$. Then, under the assumptions of Theorem 2.4 (ii), it holds that

4.18 $$\begin{aligned} \begin{aligned}&\left( \frac{1}{2\pi \textrm{i}}\right) ^2 \oint _{\gamma _1}\textrm{d}z_1\int _a^b \textrm{d}E_2 \, \textrm{e}^{\textrm{i}t(z_1-E_2-\textrm{i}\eta _2)}\frac{\eta _0}{(z_1-E_0)^2 +\eta _0^2}\langle M(z_1,E_2+\textrm{i}\eta _2)\rangle \\ =&\left( \frac{1}{2\pi \textrm{i}}\right) ^2 \oint _{\gamma _1} \textrm{d}z_1\int _a^b \textrm{d}E_2 \, \textrm{e}^{\textrm{i}t(z_1-E_2-\textrm{i}\eta _2)}\frac{\eta _0}{(z_1-E_0)^2 +\eta _0^2}\cdot \frac{\langle M_1(z_1)\rangle - \langle M_2(E_2+\textrm{i}\eta _2)\rangle }{z_1-(E_2+\textrm{i}\eta _2)-\mathfrak {s}_0^{\eta _1,\eta _2}(E_2)} \\&\qquad + \mathcal {O}\left( \eta _0+\Delta \vert \log \Delta \vert +\Delta \vert \log \mathfrak {d}\vert \right) . \end{aligned} \end{aligned}$$

Next, plugging in the Stieltjes representation $\langle M_1(z_1)\rangle = \int _{\textbf{R}}\rho _1(x)(x-z_1)^{-1}\textrm{d}x$, the $\gamma _1$ integral in Lemma 4.9 can be explicitly computed using residue calculus. The “unwanted" residue contributions arising in this way can be estimated using the oscillatory factor and integration by parts (see the proof of Lemma 4.10 in Sect. 6).

#### Lemma 4.10

(Residue computation after the first replacement) Denote $\mathfrak {a}:=\min _{E_2\in [a,b]}\vert \eta _0-\eta _2- \Im \mathfrak {s}_0^{\eta _1,\eta _2}(E_2)\vert$ and suppose that

4.19 $$\begin{aligned} \eta _1+\eta _2+\Im \mathfrak {s}_0(E_2)>0,\quad \forall E_2\in [a,b]\,. \end{aligned}$$

Then, again under the assumptions of Theorem 2.4 (ii), it holds that

4.20 $$\begin{aligned} \begin{aligned}&\left( \frac{1}{2\pi \textrm{i}}\right) ^2 \oint _{\gamma _1}\textrm{d}z_1\int _a^b \textrm{d}E_2\textrm{e}^{\textrm{i}t(z_1-E_2-i\eta _2)}\frac{\eta _0}{(z_1-E_0)^2 +\eta _0^2}\cdot \frac{\langle M_1(z_1)\rangle - \langle M_2(E_2+\textrm{i}\eta _2)\rangle }{z_1-(E_2+\textrm{i}\eta _2)-\mathfrak {s}_0^{\eta _1,\eta _2}(E_2)} \\&\quad = -\frac{1}{2\pi \textrm{i}}\int _{\textbf{R}}\textrm{d}x\int _{a}^b\textrm{d}E_2 \textrm{e}^{\textrm{i}t(x-E_2-\textrm{i}\eta _2)}\frac{\eta _0}{(x-E_0)^2+\eta _0^2}\cdot \frac{\rho _1(x)}{x-(E_2+\textrm{i}\eta _2)-\mathfrak {s}_0^{\eta _1,\eta _2}(E_2)}\\&\qquad + \mathcal {O}\left( \frac{\vert \log \mathfrak {a}\vert }{t} +\frac{\Delta + t^{-1}}{t\mathfrak {a}}+\eta _0\vert \log \mathfrak {a}\vert +\frac{\eta _0(\Delta + t^{-1})}{\mathfrak {a}}\right) . \end{aligned} \end{aligned}$$

In the following lemma, we (i) complete the integral $\int _a^b$ to a full contour integral $\oint _{\gamma _2}$, i.e., put back the tails that were cut away in Lemma 4.8, and (ii) implement the *second replacement*

4.21 $$\begin{aligned} \mathfrak {s}_0^{\eta _1, \eta _2}(E_2) \rightarrow \mathfrak {s}_0 := \mathfrak {s}_0^{\eta _1, \eta _2}\left( \left( f^{\eta _1,\eta _2}\right) ^{-1}(E_0)\right) \end{aligned}$$

inside the integral from Lemma 4.10. This replacement leads to a small error comparing to the leading term since $\mathfrak {s}_0^{\eta _1, \eta _2}(E_2)\approx \mathfrak {s}_0$ in the relevant regime $E_2\approx E_0$.

#### Lemma 4.11

(Second replacement) Let $\mathfrak {b}:=\min _{E_2\in [a,b]}\vert \eta _2+\Im \mathfrak {s}_0^{\eta _1,\eta _2}(E_2)\vert$ and $\mathfrak {s}_0$ as in (4.21). Then, again under the assumptions of Theorem 2.4 (ii), it holds that

4.22 $$\begin{aligned} \begin{aligned}&-\frac{1}{2\pi \textrm{i}}\int _{\textbf{R}} \textrm{d}x\int _{a}^b \textrm{d}E_2 \, \textrm{e}^{\textrm{i}t(x-E_2-\textrm{i}\eta _2)}\frac{\eta _0}{(x-E_0)^2+\eta _0^2}\cdot \frac{\rho _1(x)}{x-(E_2+i\eta _2+\mathfrak {s}_0^{\eta _1, \eta _2}(E_2))}\\&\quad = -\frac{1}{2\pi \textrm{i}}\int _{\textbf{R}}\textrm{d}x\oint _{\gamma _2} \textrm{d}z_2 \textrm{e}^{\textrm{i}t(x-z_2)}\frac{\eta _0}{(x-E_0)^2+\eta _0^2}\cdot \frac{\rho _1(x)}{x-(z_2+\mathfrak {s}_0)}\\&\qquad + \mathcal {O}\left( \frac{\eta _0+\mathfrak {b}}{\mathfrak {b}}\Delta \vert \log (\eta _0+\mathfrak {b})\vert +\eta _0 \vert \log \mathfrak {b}\vert +\frac{1}{t}\right) . \end{aligned} \end{aligned}$$

Armed with Lemmas 4.7–4.11, we can finally give the proof of Theorem 2.4 (ii).

#### Proof of Theorem 2.4 (ii)

Combining Lemmas 4.7 - 4.11 we find that

4.23 $$\begin{aligned} I_{E_0,\eta _0}(t) = -\frac{1}{2\pi \textrm{i}}\int _{\textbf{R}}\oint _{\gamma _2} \textrm{e}^{\textrm{i}t(x-z_2)}\frac{\eta _0}{(x-E_0)^2+\eta _0^2}\cdot \frac{\rho _1(x)\textrm{d}x}{x-(z_2+\mathfrak {s}_0)}\textrm{d}z_2 + \mathcal {O}\big (\widehat{\mathcal {E}}(t)\big ), \end{aligned}$$

where we collected all the error terms in

$$\begin{aligned} \widehat{\mathcal {E}}(t)&:=\frac{\eta _0}{\Delta }+\Delta \vert \log \Delta \vert + \Delta \vert \log \mathfrak {d}\vert + \frac{\vert \log \mathfrak {a}\vert }{t} +\frac{\Delta + t^{-1}}{t\mathfrak {a}}+\eta _0\vert \log \mathfrak {a}\vert \\&\quad +\frac{\eta _0(\Delta + t^{-1})}{\mathfrak {a}} + \frac{\eta _0+\mathfrak {b}}{\mathfrak {b}}\Delta \vert \log (\eta _0+\mathfrak {b})\vert +\eta _0 \vert \log \mathfrak {b}\vert \,. \end{aligned}$$

We shall now estimate $\widehat{\mathcal {E}}(t)$ in different time regimes. First note that Lemmas 4.4 and 4.6 imply the existence of positive constants $\lbrace c_j\rbrace _{j=1}^4$ such that

4.24 $$\begin{aligned} \begin{aligned} \vert \mathfrak {s}(z_1,z_2)\vert \le c_1\Delta ,\quad&{\text {for all}} \quad \vert z_1\vert \le 2R,\, E_2\in [a,b],\,\eta _2\in [0,1], \quad {\text {and}}\\ c_2\Delta ^2\le \Im \mathfrak {s}_0^{\eta _1,\eta _2}(E_2)\le c_3\Delta ^2,\quad&{\text {for all}} \quad E_2\in [a,b],\,\eta _j\in [0,c_4\Delta ], j=1,2. \end{aligned} \end{aligned}$$

First regime: For $1\le t\le 4Kc_3/(c_4 \Delta )$ we take $\eta _2:=8Kc_1c_3/(c_4t)$. Then, for any $E_2\in [a,b]$ it holds that

$$\begin{aligned} \eta _2+\Im \mathfrak {s}_0(E_2)\ge 8Kc_1c_3/(c_1t) - c_1\Delta \ge 4Kc_1c_3/(c_1t)>0. \end{aligned}$$

In particular, the parameters $\mathfrak {a}$, $\mathfrak {b}$, and $\mathfrak {d}$ from Lemmas 4.10, 4.11, and 4.9, respectively, are all of order 1/*t* and $\widehat{\mathcal {E}}(t)$ is bounded as

4.25 $$\begin{aligned} \widehat{\mathcal {E}}(t)\lesssim \frac{1+\log t}{t}+\frac{\eta _0}{\Delta }+\Delta \vert \log \Delta \vert +\Delta \log t,\quad {\text {for}} \quad 1\le t\le \frac{4Kc_3}{c_4}\cdot \frac{1}{\Delta }. \end{aligned}$$

Second regime: For $4Kc_3/(c_4 \Delta )\le t\le 2Kc_3/\eta _0$, we take $\eta _2:=\frac{4Kc_3}{t}$. In this regime, $\eta _2\le c_4\Delta$, so the positivity of $\eta _2+\Im \mathfrak {s}_0(E_2)$ follows from (4.24). We also have that $\eta _2\ge 2\eta _0$ and again $\mathfrak {a}\sim \mathfrak {b}\sim \mathfrak {d}\sim 1/t$. Therefore, (4.25) holds in the whole regime $1\le t\le 2Kc_3/\eta _0$.

Third regime: It remains to study the regime $2Kc_3/\eta _0 \le t \le K/\Delta ^2$. If $\eta _0\le 2c_3\Delta ^2$, it is in fact empty; hence, we may assume $\eta _0\ge 2c_3\Delta ^2$. In this case, we take $\eta _2:=\min \lbrace \eta _0/4,c_4\Delta ,1/t\rbrace$ and find that $\mathfrak {a}\sim \eta _0$, $\mathfrak {b}\gtrsim \Delta ^2$, $\mathfrak {d}\gtrsim \Delta ^2$. Moreover, the error term $\widehat{\mathcal {E}}(t)$ is bounded as

$$\begin{aligned} \widehat{\mathcal {E}}(t)\lesssim \frac{1+\log t}{t}+\Delta \vert \log \Delta \vert +\frac{\eta _0\vert \log \Delta \vert }{\Delta },\quad {\text {for}} \quad 2Kc_3/\eta _0\le t\le K/\Delta ^2. \end{aligned}$$

After having chosen $\eta _2$ in all time regimes explicitly, we can perform $z_2$-integration in (4.23). Note that in all time regimes $\eta _2$ was chosen in such a way that $\eta _2+\Im \mathfrak {s}_0>0$, which guarantees that $\gamma _2$ encircles the point $x-\mathfrak {s}_0$ for $x\in \textrm{supp}(\rho _1)$. So, (4.23) evaluates to

4.26 $$\begin{aligned} \begin{aligned}&I_{E_0,\eta _0}(t)=\textrm{e}^{\textrm{i}t\mathfrak {s}_0}\int _{\textbf{R}}\frac{\eta _0}{(x-E_0)^2+\eta _0^2}\rho _1(x)\textrm{d}x +\mathcal {O}\big (\widehat{\mathcal {E}}(t)\big ) \\&= \textrm{e}^{\textrm{i}t\mathfrak {s}_0}\Im \langle M_1(E_0+\textrm{i}\eta _0)\rangle + \mathcal {O}\big (\widehat{\mathcal {E}}(t)\big ). \end{aligned} \end{aligned}$$

After dividing by $\Im \langle M_1(E_0+\textrm{i}\eta _0)\rangle$ and taking the absolute value square, it is left to notice that, setting

4.27 $$\begin{aligned} \Gamma := 2 \, \Im \mathfrak {s}_0^{0,0}\left( \left( f^{0,0}\right) ^{-1}(E_0)\right) \,, \end{aligned}$$

it holds that

$$\begin{aligned} \Im \mathfrak {s}_0=\Im \mathfrak {s}_0^{\eta _1,\eta _2}\left( \left( f^{\eta _1,\eta _2}\right) ^{-1}(E_0)\right) = \Gamma /2 +\mathcal {O}(\Delta (\eta _1+\eta _2))= \Gamma /2+\mathcal {O}(\Delta /t). \end{aligned}$$

Here, we used (4.16) from Lemma 4.5 and (4.14) from Lemma 4.4 together with the bound $\eta _j\lesssim 1/t$, $j=1,2$. By Lemma 4.6, we finally see that the implicit constants in $\Gamma \sim \Delta ^2$ only depend on $\kappa$ and *L*. This finishes the proof of Theorem 2.4 (ii). $\square$

## Stability operator and shift: Proofs for Sect. 4.2

### Bound on the stability operator: Proof of Proposition 4.2

Throughout the proof, we will use the shorthand notations $E_j:=\Re z_j$, $\eta _j:=\vert \Im z_j\vert$, $\rho _j:=\frac{1}{\pi }\left| \langle \Im M_j(z_j)\rangle \right|$ and $\omega _j:=z_j+\langle M_j(z_j)\rangle$, for $j\in [2]$.

We will conclude Proposition 4.2 from the following lemma.

#### Lemma 5.1

Under the assumptions of Proposition 4.2 and using the notations from above, we have that:

5.1 $$\begin{aligned} \vert 1-\langle M_1M_2\rangle \vert ^{-1}&\lesssim (\eta _1/\rho _1+\eta _1/\rho _2)^{-1}\vee 1. \end{aligned}$$

5.2 $$\begin{aligned} \vert 1-\langle M_1M_2\rangle \vert ^{-1}&\lesssim (\Delta ^2 + \vert \omega _1-\overline{\omega }_2\vert ^2)^{-1}. \end{aligned}$$

5.3 $$\begin{aligned} |1 - \langle M_1 M_2 \rangle |^{-1}&\lesssim |z_1 - z_2|^{-2} \end{aligned}$$

Combining (5.1)–(5.3) with the simple observation $\vert \omega _1-\overline{\omega }_2\vert \ge \vert \langle \Im M_1\rangle +\langle \Im M_2\rangle \vert$, we conclude (4.9), i.e., the proof of Proposition 4.2. $\square$

#### Proof of Lemma 5.1

For (5.1), it is sufficient to check that for some $c\in (0,1)$ we have $\vert \langle M_1M_2\rangle \vert \le \left( 1-c(\eta _1/\rho _1+\eta _2/\rho _2)\right) \vee (1-c)$. This follows from a simple Cauchy–Schwarz inequality $\vert \langle M_1M_2\rangle \vert \le \langle |M_1|^2\rangle ^{1/2} \langle |M_2|^2\rangle ^{1/2}$ together with the estimate

$$\begin{aligned} \langle |M_j|^2\rangle ^{1/2}=\left( \frac{\langle \Im M_j\rangle }{\Im z_j+\langle \Im M_j\rangle }\right) ^{1/2}\lesssim \left( \frac{\rho _j}{\eta _j+\rho _j}\right) ^{1/2}\le \left( 1-\frac{1}{2}\cdot \frac{\eta _j}{\rho _j}\right) \vee (1-c)\,, \quad j \in [2] \end{aligned}$$

where the first step follows by taking the imaginary part of the MDE (4.6).

For (5.2), we note that it is sufficient to show

5.4 $$\begin{aligned} \Re \langle M_1M_2\rangle \le 1-c\left( \left\langle (D_1-D_2)^2\right\rangle +\vert \omega _1-\bar{\omega }_2\vert ^2\right) \quad {\text {for some}} \quad c > 0\,. \end{aligned}$$

The idea for proving (5.4) is to translate it to a question for the spectral measures of $D_1$ and $D_2$.

In order to do so, for $j \in [2]$, denote the eigenvalues and eigenvectors of $D_j$ by $\{\lambda _k^{(j)}\}_{k=1}^N$ and $\lbrace \varvec{u}_k^{(j)}\rbrace _{k=1}^N$, respectively, and the normalized spectral measure by $\mu _j:=N^{-1}\sum _{k=1}^N \delta _{\lambda _k^{(j)}}$. By the MDE (4.6), we immediately see that $\omega _j$ solves the equation $\omega _j-z_j=m_{\mu _j}(\omega _j)$, where $m_\mu (z):= \int _\textbf{R}\textrm{d}\mu (x) (x-z)^{-1}$ is the Stieltjes transform of the probability measure $\mu$. By taking the imaginary part and estimating $\vert \Im \omega _j\vert >\vert \Im \omega _j-\Im z_j\vert$ we hence find

5.5 $$\begin{aligned} \int \frac{\textrm{d}\mu _j(x)}{\vert x-\omega _j\vert ^2}<1\,. \end{aligned}$$

Using the above notations, we further see that $M_j$ can be written as $M_j=\sum _{k=1}^N (\lambda _k^{(j)}-\omega _j)^{-1}\vert \varvec{u}_k^{(j)}\rangle \langle \varvec{u}_k^{(j)}\vert$ and thus

$$\begin{aligned} \langle M_1M_2\rangle =\frac{1}{N^2}\sum _{a,b=1}^{N}\frac{1}{\lambda _a^{(1)}-\omega _1}\cdot \frac{1}{\lambda _b^{(2)}-\omega _2} f\big (\lambda _a^{(1)},\lambda _b^{(2)}\big )\,, \quad {\text {with}} \quad f\big (\lambda _a^{(1)},\lambda _b^{(2)}\big ):=N\big \vert \big \langle \varvec{u}_a^{(1)}, \varvec{u}_b^{(2)}\big \rangle \big \vert ^2\,. \end{aligned}$$

Extending *f*(*x*, *y*) to $\textbf{R}^2$ by zero, we immediately see the following properties of *f*:

1. $$f(x,y)\ge 0$$ for all $$x,y\in \mathbb {R}$$.
2. $$\int f(x,y)\textrm{d}\mu _2(y)=\textbf{1}_{\textrm{supp}\, \mu _1}(x)$$ and $$\int f(x,y)\textrm{d}\mu _1(x) = \textbf{1}_{\textrm{supp}\, \mu _2}(y)$$.
3. On $\textbf{R}^2$, $\textrm{d}\nu (x,y):=f(x,y)\textrm{d}\mu _1(x)\textrm{d}\mu _2(y)$ is a probability measure with marginals $\mu _1$ and $\mu _2$.

In this way, the desired inequality (5.4) can equivalently be rewritten as

5.6 $$\begin{aligned} \Re \iint \frac{1}{x-\omega _1}\cdot \frac{1}{y-\omega _2}\textrm{d}\nu (x,y) \le 1- c\left( \iint (x-y)^2\textrm{d}\nu (x,y)+\vert \omega _1-\bar{\omega }_2\vert ^2\right) \,. \end{aligned}$$

In this form, using (5.5), we begin by estimating the lhs. of (5.6) as

$$\begin{aligned} \Re \iint \frac{1}{x-\omega _1}\cdot \frac{1}{y-\omega _2}\textrm{d}\nu (x,y) <1-\frac{1}{2}\iint \left| \frac{1}{x-\omega _1}-\frac{1}{y-\bar{\omega }_2}\right| ^2\textrm{d}\nu (x,y), \end{aligned}$$

Thus, in order to arrive at (5.6), it suffices to bound

$$\begin{aligned} \begin{aligned}&\iint \left| \frac{1}{x-\omega _1}-\frac{1}{y-\bar{\omega }_2}\right| ^2\textrm{d}\nu (x,y)\gtrsim \iint \left| (x- y)-(\omega _1-\bar{\omega }_2)\right| ^2\textrm{d}\nu (x,y)\\&\quad = \iint (x-y)^2\textrm{d}\nu (x,y)-2\Re (\omega _1-\bar{\omega }_2)\iint (x-y)\textrm{d}\nu (x,y)+\vert \omega _1-\bar{\omega }_2\vert ^2\\&\quad =\iint (x-y)^2\textrm{d}\nu (x,y)+\vert \omega _1-\bar{\omega }_2\vert ^2. \end{aligned} \end{aligned}$$

where in the first step we employed $\vert x-\omega _1\vert \lesssim \Vert D_1\Vert +\vert \omega _1\vert \lesssim 1$ (and analogously for $\vert y-\bar{\omega }_2\vert$), while in the last step we used that fact that $D_1$ and $D_2$ are traceless. This finishes the proof of (5.2).

Finally, for (5.3), we use (5.2) and (4.11) to get that

$$\begin{aligned} \begin{aligned} \vert z_1-z_2\vert ^2&=\left| \langle M_2(D_1-D_2)M_1\rangle + (1-\langle M_1M_2\rangle )(z_1-z_2+\langle M_1\rangle - \langle M_2\rangle )\right| ^2\\&\lesssim \vert \langle M_1(D_1-D_2)M_2\rangle \vert ^2 + \vert 1-\langle M_1M_2\rangle \vert \\&\lesssim \left\langle (D_1-D_2)^2\right\rangle + \vert 1-\langle M_1M_2\rangle \vert \lesssim \vert 1-\langle M_1M_2\rangle \vert \,. \end{aligned} \end{aligned}$$

$$\square $$

### Properties of the shift: Proof of Lemmas 4.4–4.6

We finally prove the properties of the shift from Lemmas 4.4–4.6.

#### Proof of Lemma 4.4

The proof is split in two parts in the statement of the lemma.

Part (1): Given $\vert \langle M_1M_2\rangle \vert \sim 1$, note that the bound (4.13) immediately follows since, if, say, $z_1$ is such that $\rho _1(z_1) \ge \kappa /2$, then $\Vert M_1\Vert \lesssim 1$ and $\langle |M_2|^2\rangle ^{1/2}\le 1$. Both of these estimates easily follow by taking the imaginary part of the respective MDEs (4.6).

It is hence left to prove $\vert \langle M_1M_2\rangle \vert \sim 1$. The upper bound $\vert \langle M_1M_2\rangle \vert \le 1$ is a consequence of the Cauchy–Schwarz inequality and $\langle |M_j|^2\rangle ^{1/2}\le 1$. In order to prove the lower bound, we may assume w.l.o.g. that $\vert \langle M_1M_2\rangle \vert \le 1/2$, in which case $\vert 1-\langle M_1M_2\rangle \vert \sim 1$. Now, the numerator in the rhs. of the *M*-resolvent identity (4.11) is of order one, since $1\gtrsim \vert \langle M_1\rangle -\langle M_2\rangle \vert \gtrsim \vert \langle \Im M_1\rangle \vert + \vert \langle \Im M_2\rangle \vert \gtrsim 1$. Thus, by (4.11) again, we find that $\left| (z_1-z_2)\langle M_1M_2\rangle - \langle M_1(D_1-D_2)M_2\rangle \right| \sim 1$, so, in particular,

$$\begin{aligned} 1\lesssim \left| (z_1-z_2)\langle M_1M_2\rangle - \langle M_1(D_1-D_2)M_2\rangle \right| \lesssim \vert \langle M_1M_2\rangle \vert + \Delta . \end{aligned}$$

Therefore, for some constant $c>0$ which depends only on *L* and $\kappa$ we have

$$\begin{aligned} \vert \langle M_1M_2\rangle \vert \ge c-\Delta \gtrsim 1, \end{aligned}$$

i.e., we get the desired lower bound for $\vert \langle M_1M_2\rangle \vert$.

Part (2): Assume w.l.o.g. that $\rho _1(z_1) \ge \kappa /2$. The derivative $\partial _{z_1}\mathfrak {s}(z_1,z_2)$ can be computed explicitly as

$$\begin{aligned} \partial _{z_1}\mathfrak {s}(z_1,z_2) = \frac{\langle M_1^2(D_1-D_2)M_2\rangle \langle M_1M_2\rangle -\langle M_1(D_1-D_2)M_2\rangle \langle M_1^2M_2\rangle }{\langle M_1M_2\rangle ^2(1-\langle M_1^2\rangle )} \end{aligned}$$

and we note that, by analogous reasoning as in part (1), the numerator is bounded from above by $\Delta$. Since $|\langle M_1M_2\rangle |\sim 1$, from part (1), it holds that

$$\begin{aligned} \vert \partial _{z_1}\mathfrak {s}(z_1,z_2)\vert \lesssim \frac{\Delta }{\vert 1-\langle M_1^2\rangle \vert }\lesssim \Delta , \end{aligned}$$

where in the last step we used the bound $\vert 1-\langle M_1^2\rangle \vert \gtrsim \rho _1(z_1)^2$ with the aid of Proposition 4.2. $\square$

#### Proof of Lemma 4.5

The argument is split in two parts: First, we prove existence and uniqueness of the energy renormalization function *f*. Second, we estimate the partial derivatives (4.16) of *f* and the renormalized (one point) shift $\mathfrak {s}_0$.

$\underline{\text {Part}\,\, \text {(1):}\,\, \text {Existence}\,\, \text {and}\,\, \text {uniqueness}\,\, \text {of}\,\, f.}$ First, from Lemma 4.5, we have that, for $z_1$ with $|z_1| \le L$ and $\Im z_1 < 0$, it holds that $\vert \mathfrak {s}(z_1,z_2)\vert \le C\Delta$ for some $C > 0$. For fixed $z_2 = E_2 + \textrm{i}\eta _2$, we introduce the auxiliary (differentiable) function

$$\begin{aligned} h(E_1):=E_1-E_2-\Re \mathfrak {s}(E_1-\textrm{i}\eta _1,E_2+\textrm{i}\eta _2)\,, \end{aligned}$$

which has the property that $h(E_1)<0$ for $E_1< E_2-C\Delta$, and $h(E_2)>0$ for $E_1>E_2+C\Delta$. Hence, $h(E_1)=0$ has a solution in $\mathcal {I}:= [E_2-C\Delta ,E_2+C\Delta ]$. To see uniqueness, we differentiate *h* and find that $h'(E_1) \ge 1 - c \Delta$ for $E_1\in \mathcal {I}$ and some $c > 0$ by means of (4.14) from Lemma 4.4. Thus, *h* has a unique zero on $\mathcal {I}$ (and hence in $(-L,L)$) which we denote by $f(E_2) = f^{\eta _1, \eta _2}(E_2)$—the desired energy renormalization function. Differentiability of *f* easily follows from the implicit function theorem.

Part (2): Bounds on derivatives. Differentiating the identity $h(f^{\eta _1, \eta _2}(E_2))=0$ in $E_2$, we find that

$$\begin{aligned} \partial _{E_2} f^{\eta _1, \eta _2}(E_2) = \frac{1+\Re \partial _2 \mathfrak {s}(f(E_2)-\textrm{i}\eta _1,E_2+\textrm{i}\eta _2)}{1-\Re \partial _1\mathfrak {s}(f(E_2)-\textrm{i}\eta _1,E_2+\textrm{i}\eta _2))} = 1 +\mathcal {O}(\Delta ), \end{aligned}$$

by means of (4.14) from Lemma 4.4. Here, $\partial _j \mathfrak {s}$ denotes the partial derivative of $\mathfrak {s}$ w.r.t. its $j^\textrm{th}$ argument. Similarly,

$$\begin{aligned} \partial _{\eta _1}f^{\eta _1,\eta _2}(E_2)=-\frac{\Re \left[ \textrm{i}\partial _1\mathfrak {s}\right] }{1-\Re \left[ \partial _1\mathfrak {s}\right] },\quad \partial _{\eta _2}f^{\eta _1,\eta _2}(E_2)=\frac{\Re \left[ \textrm{i}\partial _1\mathfrak {s}\right] }{1-\Re \left[ \partial _2\mathfrak {s}\right] }\,, \end{aligned}$$

where $\mathfrak {s}$ has arguments $f(E_2)-\textrm{i}\eta _1$ and $E_2+\textrm{i}\eta _2$. This concludes the bound $\left| \partial _{\eta _j} f^{\eta _1,\eta _2}(E_2)\right| \lesssim \Delta$ for $j=1,2$. The bound on $\vert \partial _{E_2}\mathfrak {s}_0(E_2)\vert$ is obtained in a similar fashion and thus left to the reader. $\square$

#### Proof of Lemma 4.6

The proof is divided in two parts: In the first part, we prove (4.17) for $\eta _1=\eta _2=+0$. In the second part of the argument, we treat the general case as a perturbation thereof.

Part (1): Proof on the real line. Applying the *M*-resolvent identity (4.11) for $z_1:=f(E)-\textrm{i}0$ and $z_2:=E+\textrm{i}0$ and using Proposition 4.2, we find that

$$\begin{aligned} \left| \frac{\langle M_1(z_1)\rangle - \langle M_2(z_2)\rangle }{f(E)-E-\mathfrak {s}_0(E)}\right| \lesssim \frac{1}{\Delta ^2}\,. \end{aligned}$$

Since the numerator on the lhs. is of order one and the real part of the denominator vanishes by definition of *f*(*E*), we deduce that

$$\begin{aligned} \Delta ^2\lesssim \left| \Im \left[ f(E)-E-\mathfrak {s}_0(E)\right] \right| = \vert \Im \mathfrak {s}_0(E)\vert \,, \end{aligned}$$

i.e., we have a lower bound on the *modulus* of $\Im \mathfrak {s}_0(E)$. To turn this into a lower bound on $\Im \mathfrak {s}_0(E)$ itself, we need to show that it is positive.

This will be done via a proof by contraction: Suppose that $\Im \mathfrak {s}_0(E)<0$. By (4.11) for $z_1:=f(E)-\textrm{i}0$, $z_2:=E+\textrm{i}0$ we get

5.7 $$\begin{aligned} \frac{\langle M_1M_2\rangle }{1-\langle M_1M_2\rangle } = \frac{\langle M_1\rangle - \langle M_2\rangle }{-\textrm{i}\Im \mathfrak {s}_0(E)}\,. \end{aligned}$$

Since $\Im \left[ \langle M_1\rangle - \langle M_2\rangle \right] = -c$ for some $c > 0$ and $|\Re \left[ \langle M_1\rangle - \langle M_2\rangle \right] | \lesssim \Delta$, we obtain, using our assumption $\Im \mathfrak {s}_0(E)<0$,

$$\begin{aligned} \langle M_1\rangle - \langle M_2\rangle = \vert \langle M_1\rangle -\langle M_2\rangle \vert \textrm{e}^{-\frac{\textrm{i}\pi }{2}+\textrm{i}\mathcal {O}(\Delta )}\quad {\text {and}}\quad \frac{\langle M_1\rangle - \langle M_2\rangle }{-\textrm{i}\Im \mathfrak {s}_0(E)}= \left| \frac{\langle M_1\rangle - \langle M_2\rangle }{-\textrm{i}\Im \mathfrak {s}_0(E)}\right| \textrm{e}^{\textrm{i}\pi +\textrm{i}\mathcal {O}(\Delta )}\,, \end{aligned}$$

where here and in the following $\mathcal {O}(\Delta )$ is real-valued. In a similar way, we find that $\langle M_1M_2\rangle =1+\mathcal {O}(\Delta ) + \textrm{i}\mathcal {O}(\Delta )$ and $\langle M_1M_2\rangle = \vert \langle M_1M_2\rangle \vert \textrm{e}^{\textrm{i}\mathcal {O}(\Delta )}$. Hence, (5.7) implies

$$\begin{aligned} 1-\langle M_1M_2\rangle = \left| \frac{-\textrm{i}\Im \mathfrak {s}_0(E)}{\langle M_1\rangle - \langle M_2\rangle }\langle M_1M_2\rangle \right| \textrm{e}^{\textrm{i}\pi + \mathcal {O}(\Delta )}\,, \end{aligned}$$

i.e., in particular, $\Re \left[ 1-\langle M_1M_2\rangle \right] <0$. On the other hand, it holds that $\Re \left[ 1-\langle M_1M_2\rangle \right] \ge 1-\vert \langle M_1M_2\rangle \vert \ge 0$, so we arrived at a *contradiction* and thus $\Im \mathfrak {s}_0(E) > 0$ and $\Im \mathfrak {s}_0(E)\gtrsim \Delta ^2$.

For part (1), we are now left to prove $\vert \Im \mathfrak {s}_0(E)\vert \lesssim \Delta ^2$, which is done via a perturbative argument in Appendix A. This concludes part (1), i.e., $\Im \mathfrak {s}_0^{0,0}(E) \sim \Delta ^2$.

Part (2): Extension away from the real line. By (4.16) and the fundamental theorem of calculus, we have

$$\begin{aligned} \left| \Im \mathfrak {s}_0^{\eta _1,\eta _2}(E)-\Im \mathfrak {s}_0^{0,0}(E)\right| \le \left| \int \limits _0^{\eta _1} \partial _{\zeta _1} \mathfrak {s}_0^{\zeta _1,\eta _2}(E)\textrm{d}\zeta _1\right| + \left| \int \limits _0^{\eta _2} \partial _{\zeta _2} \mathfrak {s}_0^{0,\zeta _2}(E)\textrm{d}\zeta _2\right| \lesssim \Delta (\eta _1+\eta _2)\,. \end{aligned}$$

Hence, if $0 <\eta _j \le c_2 \Delta$ for some $c_2 > 0$ small enough, we obtain $\Im \mathfrak {s}_0^{\eta _1,\eta _2}(E)\sim \Im \mathfrak {s}_0^{0,0}(E)\sim \Delta ^2$. $\square$

## Contour integration: Proof of technical lemmas from Sect. 4.3

The goal of this section is to give the proofs of the technical lemmas from Sect. 4.3, for which we recall the construction of the contours $\gamma _1, \gamma _2$ from Sect. 4, in particular (4.2)–(4.3) and Fig. 1, and the definition of the [*a*, *b*] interval from the beginning of Sect. 4.3.

In all of the estimates below, we will frequently use the following simple tools:

- To gain 1/*t*-factors from the oscillatory $\textrm{e}^{\textrm{i}t (z_1 -z_2)}$, we integrate by parts.
- When pulling absolute values inside an integral, we bound $|\textrm{e}^{\textrm{i}t (z_1 -z_2)}| \lesssim 1$ (recall $|\Im z_j| \lesssim 1/t$).
- The convolution of two Cauchy kernels yields another Cauchy kernel: For $\eta _j > 0$ and $E_j \in \textbf{R}$, $j\in [2]$ it holds that
  6.1 $$\begin{aligned} \int _\textbf{R}\frac{\eta _1}{(x-E_1)^2 + \eta _1^2} \frac{\eta _2}{(x-E_2)^2 + \eta _2^2} \textrm{d}x \lesssim \frac{\eta _1+\eta _2}{(E_1-E_2)^2 + (\eta _1 + \eta _2)^2} \,. \end{aligned}$$

We now turn to the proofs of the lemmas from Sect. 4.3.

### The second line of (4.23) is negligible: Proof of Lemma 4.7

We discuss the contributions from the flat and semicircular part of $\gamma _2$ separately (recall (4.3)).

First, the smallness of the integral over $\gamma _2^{(2)}$ (the semicircular part) is granted by the factor $\textrm{e}^{t\Im z_2}$ (note that $\Im z_2 \in [-R + \eta _2, \eta _2]$) and the estimate $|\langle M(E_0+i\eta _0,z_2)\rangle |\lesssim 1$, which follows from (4.9). More precisely, we have that

6.2 $$\begin{aligned} \left| \oint _{\gamma _2^{(2)}} \textrm{e}^{\textrm{i}t(E_0+\textrm{i}\eta _0-z_2)}\left\langle M(E_0+\textrm{i}\eta _0,z_2)\right\rangle \textrm{d}z_2\right| \lesssim R\int _{\pi }^{2\pi } \textrm{e}^{tR\sin \theta }\textrm{d}\theta \lesssim \frac{1}{t}\,. \end{aligned}$$

Next, we bound the integral over $\gamma _2^{(1)}$—the flat part. As a first step, integration by parts yields

$$\begin{aligned}  &   \left| \int _{\gamma _2^{(1)}} \textrm{e}^{\textrm{i}t(E_0+\textrm{i}\eta _0-z_2)}\left\langle M(E_0+\textrm{i}\eta _0,z_2)\right\rangle \textrm{d}z_2\right| \\  &   \quad \lesssim \frac{1}{t} +\left| \frac{1}{\textrm{i}t}\int _{-R}^R \textrm{e}^{-\textrm{i}tE_2}\partial _{E_2}\left\langle M(E_0+\textrm{i}\eta _0,E_2+\textrm{i}\eta _2)\right\rangle \textrm{d}E_2\right| . \end{aligned}$$

The derivative can be explicitly computed as

6.3 $$\begin{aligned} \partial _{z_2}\langle M(z_1,z_2)\rangle =\frac{\langle M_1M_2^2\rangle }{(1-\langle M_2^2\rangle )(1-\langle M_1M_2\rangle )^2}. \end{aligned}$$

Since $E_0$ is in the bulk of $\rho _1$ and $z_0:=E_0+\textrm{i}\eta _0$ and $z_2$ are in the same half-plane we infer $\vert 1-\langle M_1M_2\rangle \vert \gtrsim 1$ and thus

$$\begin{aligned} \left| \oint _{\gamma _2^{(1)}} \textrm{e}^{\textrm{i}t(E_0+i\eta _0-z_2)}\left\langle M(E_0+\textrm{i}\eta _0,z_2)\right\rangle \textrm{d}z_2\right| \lesssim \frac{1}{t} + \frac{1}{t}\int _{-R}^R \left| \frac{1}{1-\langle M_2(E_2+\textrm{i}\eta _2)^2\rangle }\right| \textrm{d}E_2. \end{aligned}$$

In order to conclude the proof of Lemma 4.7, we finally use that the one-body stability operator $\vert 1-\langle M_2(E_2+\textrm{i}\eta _2)^2\rangle \vert ^{-1}$ is locally integrable, see Lemma A.1 in Appendix A. $\square$

### Cutting tails in the first line of (4.23): Proof of Lemma 4.8

For cutting the tails, we focus on the more critical regime, where both parameters are on the horizontal part of the contours, $z_j \in \gamma _j^{(1)}$ for $j \in [2]$ (recall (4.2)–(4.3)). Indeed, if this is not the case, a simple computation using Proposition 4.2 and arguing similarly to (6.2) yields $(1+\eta _0/\Delta )/t\lesssim 1/t$ as an upper bound for the corresponding integrals.

In the critical regime $z_j \in \gamma _j^{(1)}$ for $j \in [2]$ we carry out only the case $E_2 = \Re z_2 \in [b,R]$; for $E_2\in [-R,a]$ the argument is identical. Let $\delta :=(b-E_0)/2$ and split the region of the $E_1 = \Re z_1$-integration into the two parts, $[b-\delta ,2R]$ and $[-2R,b-\delta ]$. In the first regime, using $|E_1- E_0| \gtrsim 1$ and, from Proposition 4.2, $|\langle M(E_1-\textrm{i}\eta _1,E_2+\textrm{i}\eta _2)\rangle | \lesssim \big ((E_1-E_2)^2+\Delta ^2\big )^{-1}$, we find that

$$\begin{aligned} \begin{aligned} \int _{b-\delta }^{2R}\int _b^R \left| \frac{\eta _0}{(E_1-\textrm{i}\eta _1-E_0)^2 +\eta _0^2}\langle M(E_1-\textrm{i}\eta _1,E_2+\textrm{i}\eta _2)\rangle \right| \textrm{d}E_1\textrm{d}E_2\lesssim \frac{\eta _0}{\Delta }. \end{aligned} \end{aligned}$$

For $E_1\in [-2R,b-\delta ]$, by Proposition 4.2 again, we have $\vert \langle M(z_1,z_2)\rangle \vert \lesssim 1$, since $\vert E_1-E_2\vert \sim 1$. Using this and integration by parts in $E_2$, similarly to the proof of Lemma 4.7, in combination with (6.3) and Lemma A.1, we find that

$$\begin{aligned} \left| \int _{-2R}^{b-\delta }\int _b^R \textrm{e}^{\textrm{i}t(z_1-E_2-\textrm{i}\eta _2)}\frac{\eta _0}{(E_1-\textrm{i}\eta _1-E_0)^2 +\eta _0^2}\langle M(E_1-\textrm{i}\eta _1,E_2+\textrm{i}\eta _2)\rangle \textrm{d}E_1\textrm{d}E_2\right| \lesssim \frac{1}{t} \,. \end{aligned}$$

This finishes the proof of Lemma 4.8. $\square$

### First replacement: Proof of Lemma 4.9

Let $\delta > 0$ be such that $[a-\delta ,b+\delta ]$ is in the bulk of $\rho _1$. We now compare the two integrals on the lhs. and rhs. of (4.18) by taking their difference. Using integration by parts, the contribution from $(z_1, z_2) \in \gamma _1^{(2)}\times [a,b]$ is bounded by $\eta _0/t$. Analogously to the proof of Lemma 4.8, we also find that the contribution from $([-R,R]{\setminus } [a-\delta ,b+\delta ])\times [a,b]$ is bounded by $\eta _0$, since in this regime $\vert \langle M(z_1,I,z_2)\rangle \vert \lesssim 1$ and $\vert z_1-z_2-\mathfrak {s}_0^{\eta _1,\eta _2}(E_2)\vert ^{-1}\gtrsim 1$. From now on and until the end of Sect. 6.5 we use the shorthand notation $\mathfrak {s}_0(E_2):=\mathfrak {s}_0^{\eta _1,\eta _2}(E_2)$.

We are hence left to estimate the contribution from the region $[a-\delta ,b+\delta ]\times [a,b]$. Using that $\vert \mathfrak {s}(z_1,z_2)-\mathfrak {s}_0(E_2)\vert \lesssim \Delta \vert E_1-\Re f(z_2)\vert$ by means of Lemma 4.4, we find that this can be bounded by

$$\begin{aligned} \mathcal {E}:= \int _{a-\delta }^{b+\delta }\int _a^b \frac{\eta _0}{(E_1-E_0)^2+\eta _0^2} \cdot \frac{\Delta \vert E_1-\Re f(z_2)\vert }{\vert z_1-z_2-\mathfrak {s}(z_1,z_2)\vert \cdot \vert z_1-z_2-\mathfrak {s}_0(E_2)\vert }\textrm{d}E_1\textrm{d}E_2\,. \end{aligned}$$

To have better control on $\mathcal {E}$, we now bound the denominators in the second factor from below. First, using the definition of $\mathfrak {d}$ from the formulation of Lemma 4.9, we get

6.4 $$\begin{aligned} \vert z_1-z_2-\mathfrak {s}_0(E_2)\vert ^2 = (E_1-E_2-\Re \mathfrak {s}_0(E_2))^2 + (\eta _1+\eta _2+\Im \mathfrak {s}_0(E_2))^2\gtrsim (E_1-f(E_2))^2+\mathfrak {d}^2\,. \end{aligned}$$

Next, using that $\vert z_1-z_2-\mathfrak {s}(z_1,z_2)\vert \gtrsim \Delta ^2$, as simple consequence of the stability bound (4.9), we infer

6.5 $$\begin{aligned} \vert z_1-z_2-\mathfrak {s}(z_1,z_2)\vert ^2 \sim \vert z_1-z_2-\mathfrak {s}(z_1,z_2)\vert ^2 +\Delta ^4\gtrsim (E_1-E_2-\Re \mathfrak {s}(z_1,z_2))^2 + \Delta ^4\,. \end{aligned}$$

Finally, using the defining properties of the renormalization function *f* given in Lemma 4.5, (4.14) from Lemma 4.4, and the triangle inequality, one easily sees that

6.6 $$\begin{aligned} \vert E_1-E_2-\Re \mathfrak {s}(z_1,z_2)\vert \sim \vert E_1-f(E_2)\vert . \end{aligned}$$

Hence, combining (6.4) and (6.5)–(6.6) we find that

$$\begin{aligned} \begin{aligned} \mathcal {E}&\lesssim \int _{a-\delta }^{b+\delta }\int _a^b \frac{\eta _0}{(E_1-E_0)^2+\eta _0^2}\cdot \frac{\Delta \vert E_1-f(E_2)\vert }{\vert E_1-f(E_2)\vert ^2 +(\min \lbrace \mathfrak {d},\Delta ^2\rbrace )^2}\textrm{d}E_1\textrm{d}E_2\\&\lesssim \int _{a-\delta }^{b+\delta }\frac{\eta _0\Delta (\vert \log \Delta \vert +\vert \log \mathfrak {d}\vert )}{(E_1-E)^2+\eta _0^2}\textrm{d}E_1 \lesssim \Delta (\vert \log \Delta \vert +\vert \log \mathfrak {d}\vert ). \end{aligned} \end{aligned}$$

where in the second step we changed the integration variable from $E_2$ to $f(E_2)$ and employed (4.16) from Lemma 4.5. This concludes the proof of Lemma 4.9. $\square$

### Residue computation after the first replacement: Proof of Lemma 4.10

Using the integral representation $\langle M_1(z_1)\rangle = \int _{\textbf{R}}\rho _1(x)(x-z_1)^{-1}\textrm{d}x$ and carrying out the residue computation (note that (4.19) ensures $z_2+\mathfrak {s}_0(E_2)$ is encircled by the contour $\gamma _1$), we find the lhs. of (4.20) to equal

$$\begin{aligned} -\frac{1}{2 \pi \textrm{i}} \int _a^b \textrm{d}E_2 \int _{\textbf{R}} \textrm{e}^{\textrm{i}t(x-E_2 - \textrm{i}\eta _2 )}\frac{\eta _0}{(x-E_0)^2+\eta _0^2}\cdot \frac{\rho _1(x)\textrm{d}x}{x-(E_2 + \textrm{i}\eta _2 +\mathfrak {s}_0(E_2))} + \mathcal {E}_1 + \mathcal {E}_2 \,, \end{aligned}$$

where we introduced the shorthand notations

$$\begin{aligned} \begin{aligned}&\mathcal {E}_1:=- \frac{1}{4\pi }\int _a^b \textrm{e}^{\textrm{i}t(E_0+\textrm{i}\eta _0-E_2-\textrm{i}\eta _2)}\frac{\langle M_1(E_0+\textrm{i}\eta _0)\rangle - \langle M_2(E_2+\textrm{i}\eta _2)\rangle }{E_0+\textrm{i}\eta _0-(E_2+\textrm{i}\eta _2+\mathfrak {s}_0(E_2))}\textrm{d}E_2,\\&\mathcal {E}_2:=\frac{1}{2\pi \textrm{i}}\int _a^b \textrm{e}^{\textrm{i}t\mathfrak {s}_0(E_2)} \frac{\eta _0\left( \langle M_1(E_0+\textrm{i}\eta _0)\rangle -\langle M_2(E_2+\textrm{i}\eta _2)\rangle \right) }{(E_2+\textrm{i}\eta _2+\mathfrak {s}_0(E_2)-E_0)^2 +\eta _0^2}\textrm{d}E_2\,. \end{aligned} \end{aligned}$$

Moreover, we shall abbreviate $z_0:=E_0+\textrm{i}\eta _0$, $z_2:=E_2+\textrm{i}\eta _2$. Then, to estimate $\mathcal {E}_1$, we employ integration by parts and find that since $\left| \partial _{E_2}\langle M_2(z_2)\rangle \right| \lesssim 1$ as $\rho _2(z_2) \gtrsim 1$, using (4.16) from Lemma 4.5, and recalling the definition of $\mathfrak {a}$ from the formulation of Lemma 4.10,

$$\begin{aligned} \left| \partial _{E_2}\frac{\langle M_1(z_0)\rangle - \langle M_2(z_2)\rangle }{z_0-(z_2+\mathfrak {s}_0(E_2))}\right| \lesssim \frac{1}{\vert E_0- f(E_2)\vert + \mathfrak {a}} + \frac{\vert \langle M_1(z_0)\rangle - \langle M_2(z_2)\rangle \vert }{\vert E_0- f(E_2)\vert ^2 + \mathfrak {a}^2}. \end{aligned}$$

Applying the *M*-resolvent identity (4.11) to $z_0$ and $z_2$ we infer, by application of the stability bound from Proposition 4.2 together with (4.13) and $\eta _2 \lesssim 1/t$, $\eta _0 \lesssim \Delta$, that $\vert \langle M_1(z_0)\rangle - \langle M_2(z_2)\rangle \vert \lesssim \vert E_0- f(E_2)\vert + \Delta + 1/t$, and hence

$$\begin{aligned} \begin{aligned} \vert \mathcal {E}_1\vert&\lesssim \frac{1}{t}+\frac{1}{t}\int _a^b \left( \frac{1}{\vert E_0-f(E_2)\vert + \mathfrak {a}}+\frac{\vert E_0-f(E_2)\vert + \Delta + t^{-1}}{\vert E_0- f(E_2)\vert ^2 + \mathfrak {a}^2}\right) \textrm{d}E_2\\&\lesssim \frac{\vert \log \mathfrak {a}\vert }{t}+\frac{\Delta + t^{-1}}{t\mathfrak {a}}. \end{aligned} \end{aligned}$$

Similarly, $\mathcal {E}_2$ admits the bound $\vert \mathcal {E}_2\vert \lesssim \eta _0\vert \log \mathfrak {a}\vert +\eta _0 \mathfrak {a}^{-1} (\Delta + t^{-1})$. This finishes the proof of Lemma 4.10. $\square$

### Second replacement: Proof of Lemma 4.11

The argument is split in two parts. First, we estimate the error of the second replacement within the interval [*a*, *b*]. Then, we put back the tails to complete the full contour integral.

For the first part, using $\vert \mathfrak {s}_0(E_2)-\mathfrak {s}_0\vert \lesssim \Delta \vert f(E_2)-E_0\vert$ as a consequence of (4.16), we find the error to be bounded by (a constant times) $\mathcal {E}_1 + \mathcal {E}_2$, where

6.7 $$\begin{aligned} \mathcal {E}_1:=\int _{\textbf{R}} \textrm{d}x \int _a^b \frac{\eta _0}{(x-E_0)^2+\eta _0^2}\cdot \frac{\Delta \vert f(E_2)-E_0\vert }{\vert x-(z_2+\mathfrak {s}_0(E_2))\vert ^2}\textrm{d}E_2 \end{aligned}$$

and $\mathcal {E}_2$ is the same integral as $\mathcal {E}_1$, but with $\mathfrak {s}_0(E_2)$ being replaced by $\mathfrak {s}_0$. Next, convolving Cauchy kernels (6.1) in the *x*-variable and using (4.16) together with the definition of $\mathfrak {b}$ we arrive at

$$\begin{aligned} \mathcal {E}_1\lesssim \frac{\eta _0+\mathfrak {b}}{\mathfrak {b}}\int _a^b \frac{\Delta \vert f(E_2)-E_0\vert }{(f(E_2)-E_0)^2 + (\eta _0+\mathfrak {b})^2}\textrm{d}E_2\lesssim \frac{\eta _0+\mathfrak {b}}{\mathfrak {b}} \Delta \vert \log (\eta _0+\mathfrak {b})\vert \,. \end{aligned}$$

For $\mathcal {E}_2$, the argument is similar: We simply replace $f(E_2) - E_0$ in the denominator by $E_0 - E_2 - \Re \mathfrak {s}_0$ and estimate $\vert E_0- f(E_2)\vert \lesssim \vert E_0-E_2-\Re \mathfrak {s}_0\vert$ in the numerator. This shows that the error for the first bound is bounded by $(\eta _0+\mathfrak {b})\mathfrak {b}^{-1}\Delta \vert \log (\eta _0+\mathfrak {b})\vert$.

In the second part, we estimate the tails on the rhs. of (4.22). In the regime when $z_2\in \gamma _2^{(2)}$ we find the bound 1/*t*, similarly to (6.2). If instead $z_2\in \gamma _2^{(1)}\setminus ([a,b]+\textrm{i}\eta _2)$, say, $E_2 = \Re z_2 \in [b,R]$ for concreteness, we have that $\vert E_0-E_2-\Re \mathfrak {s}_0\vert \sim 1$, so the singularities in *x* on the rhs. of (4.22) are separated from each other. Now, pick $\delta \sim 1$ such that $[E_0-\delta ,E_0+\delta ]\subset [a,b]$ and $\vert x-E_2-\Re \mathfrak {s}_0\vert \sim 1$ for any $E_2\in [b,R]$, $x\in [E_0-\delta ,E_0+\delta ]$. Then, for $\vert x-E_0\vert \ge \delta$, it holds that

$$\begin{aligned} \left| \int _{\vert x-E_0\vert \ge \delta }\textrm{d}x\int _b^R e^{it(x-E_2-i\eta _2)}\frac{\eta _0}{(x-E_0)^2+\eta _0^2}\cdot \frac{\rho _1(x)\textrm{d}x}{x-(E_2+i\eta _2+\mathfrak {s}_0)}\textrm{d}E_2\right| \lesssim \eta _0\vert \log \mathfrak {b}\vert \,, \end{aligned}$$

where, in order to get $\mathfrak {b}$, we employed Lemma 4.6 and (4.16). Finally, for $\vert x-E_0\vert \le \delta$, we employ integration by parts in $E_2$ and use $\vert x-E_2-\Re \mathfrak {s}_0\vert \sim 1$ for any $E_2\in [b,R]$, $x\in [E_0-\delta ,E_0+\delta ]$ to get

$$\begin{aligned} \left| \int _{E_0-\delta }^{E_0+\delta } \textrm{d}x \textrm{e}^{\textrm{i}t(x-\textrm{i}\eta _2)}\frac{\eta _0 \, \rho _1(x)}{(x-E_0)^2+\eta _0^2}\int _b^R \textrm{e}^{-\textrm{i}tE_2}\frac{\textrm{d}E_2}{x-(E_2+i\eta _2+\mathfrak {s}_0)}\right| \lesssim \frac{1}{t}\,. \end{aligned}$$

This finishes the justification of the replacement (4.22) and thus the proof of Lemma 4.11. $\square$

## Second echo protocol: Proof of Theorem 2.10

The argument for part (i) is very similar to that for the proof of Theorem 2.4 (i). The only two differences are the following: First, the formerly algebraic cancellations $\langle \widetilde{P} [H_1, H_2] \rangle = 0$ below (3.3) and (3.5) are replaced by the estimate $|\langle \psi , W \phi \rangle | \prec \Vert \psi \Vert \, \Vert \phi \Vert N^{-1/2}$ for deterministic $\phi , \psi \in \textbf{C}^N$. This follows by residue calculus and using an isotropic global law for the Wigner matrix *W* together with the fact that the first moment of the semicircular density vanishes, $\int _\textbf{R}x \rho _{\textrm{sc}}(x) \textrm{d}x = 0$, by symmetry. More precisely, using $\Vert W \Vert \le 2+ \epsilon$ with very high probability,

7.1 $$\begin{aligned} \begin{aligned} |\langle \psi , W \phi \rangle |&= \left| \frac{1}{2 \pi \textrm{i}} \oint _{|z|=3} z \langle \psi , (W-z)^{-1} \phi \rangle \textrm{d}z \right| \\&\lesssim \Vert \psi \Vert \, \Vert \phi \Vert \left| \frac{1}{2 \pi \textrm{i}} \oint _{|z|=3} z m_{\textrm{sc}}(z) \textrm{d}z \right| + \Vert \psi \Vert \, \Vert \phi \Vert \mathcal {O}_\prec (N^{-1/2}) \\&\lesssim \Vert \psi \Vert \, \Vert \phi \Vert \left| \int _\textbf{R}x \rho _{\textrm{sc}}(x) \textrm{d}x \right| + \Vert \psi \Vert \, \Vert \phi \Vert \mathcal {O}_\prec (N^{-1/2}) \prec \Vert \psi \Vert \, \Vert \phi \Vert N^{-1/2} \end{aligned} \end{aligned}$$

where, to go to the last line, we used the Stieltjes representation $m_{\textrm{sc}}(z) = \int _\textbf{R}(x-z)^{-1} \rho _{\textrm{sc}}(x) \textrm{d}x$ and simple residue calculus. Second, in the analog of (3.6) it suffices to estimate all the $\lambda W$ simply by operator norm, recalling $\Vert W \Vert \le 2+ \epsilon$ with very high probability. The rest of the argument goes along the same lines as in the proof of Theorem 2.4 (i) with straightforward modifications.

Part (ii) may be derived from [22, Theorem 2.4], but here we give a direct proof relying just on the argument given in [22, Section 3.2.1]. First, by means of the single resolvent global law, we have that

7.2 $$\begin{aligned} \begin{aligned} \langle \psi _0, \textrm{e}^{\textrm{i}t H_\lambda } \textrm{e}^{- \textrm{i}t H_0} \psi _0 \rangle&= \frac{1}{2 \pi \textrm{i}} \oint _{\gamma } \textrm{e}^{\textrm{i}t z}\langle \psi _0, G_\lambda (z) \textrm{e}^{- \textrm{i}t H_0} \psi _0 \rangle \textrm{d}z \\&= \frac{1}{2 \pi \textrm{i}} \oint _{\gamma } \textrm{e}^{\textrm{i}t z}\langle \psi _0, M_\lambda (z) \textrm{e}^{- \textrm{i}t H_0} \psi _0 \rangle \textrm{d}z + \mathcal {O}_\prec \big (C(t, \lambda )/\sqrt{N}\big ) \end{aligned} \end{aligned}$$

for some constant $C(t, \lambda ) > 0$ depending only on time *t* and coupling $\lambda$. Next, we approximate $\langle M_\lambda (z) \rangle \approx \overline{m_0(E_0)}$, leading to

7.3 $$\begin{aligned} M_\lambda (z) \approx \frac{1}{H_0 -z - \lambda ^2 \overline{m_0(E_0)}}\,. \end{aligned}$$

Plugging the approximation (7.3) into (7.2), we find

7.4 $$\begin{aligned} \frac{1}{2 \pi \textrm{i}} \oint _{\gamma } \textrm{e}^{\textrm{i}t z}\big \langle \psi _0, \big (H_0 -z - \lambda ^2 \overline{m_0(E_0)}\big )^{-1} \textrm{e}^{- \textrm{i}t H_0} \psi _0 \big \rangle \textrm{d}z = \textrm{e}^{- \textrm{i}\overline{m_0(E_0)} \lambda ^2 t} \end{aligned}$$

from simple residue calculus for $\lambda >0$ small enough, using that $|m_0(E_0)| \lesssim 1$ (as follows from $\rho _0$ being $C^{1,1}$ around $E_0$; recall (2.18)) and $\gamma$ encircles the spectrum of $H_0$. We have thus extracted the main term in (7.2), and it remains to estimate the errors resulting from the replacements in (7.3).

Denoting the spectral decomposition of $H_0$ by $H_0 = \sum _j \mu _j \mathinner {|{\varvec{u}_j}\rangle } \mathinner {\langle {\varvec{u}_j}|}$ and using Assumption 2.9, we have that

7.5 $$\begin{aligned} \frac{1}{2 \pi \textrm{i}} \oint _{\gamma } \textrm{e}^{\textrm{i}t z}\langle \psi _0, M_\lambda (z) \textrm{e}^{- \textrm{i}t H_0} \psi _0 \rangle \textrm{d}z = \sum _{\mu _j \in I_\Delta } \langle \psi _0, \varvec{u}_j \rangle \langle \varvec{u}_j, \psi _t \rangle \widetilde{\vartheta }(j) \,, \end{aligned}$$

where we denoted $\psi _t:= \textrm{e}^{-\textrm{i}t H_0} \psi _0$ and

7.6 $$\begin{aligned} \widetilde{\vartheta }(j) := \frac{1}{2 \pi \textrm{i}} \oint _{\gamma } \frac{\textrm{e}^{\textrm{i}t z}}{\mu _j - z - \lambda ^2 \langle M_\lambda (z) \rangle } \textrm{d}z\,. \end{aligned}$$

The key to approximating (7.5) is the following lemma, the proof of which is identical to that of [22, Lemma 3.3] and so omitted.

### Lemma 7.1

(cf. Lemma 3.3 in [22]) Under the above assumptions and notations, for every $j \in [N]$ such that $\mu _j \in I_\Delta$, denote

$$\begin{aligned}\vartheta (j):= (2 \pi \textrm{i})^{-1} \oint _{\gamma } \textrm{e}^{\textrm{i}t z}(\mu _j - z - \lambda ^2 \overline{m_0(E_0)})^{-1} \textrm{d}z. \end{aligned}$$

Then, it holds that

7.7 $$\begin{aligned} \sup _{\mu _j \in I_\Delta } \left| \widetilde{\vartheta }(j) - \vartheta (j) \right| \lesssim \mathcal {E} \end{aligned}$$

for sufficiently small $\lambda > 0$ and *N* large enough (dependent on $\lambda$, cf. [22, Lemma A.1]). Here, recalling (2.16) for the definition of $\epsilon _0 = \epsilon _0(N)$, we denoted

7.8 $$\begin{aligned} {\mathcal {E}} = \mathcal {E}(\lambda , t, \Delta , N):= \lambda ^2 t \, \Delta + \lambda \, (1 + \lambda ^2 t)+ \frac{\lambda }{\Delta }\left( 1 + \frac{\lambda }{\Delta }\right) + \lambda ^2 t \, \epsilon _0\,. \end{aligned}$$

Therefore, by means of Lemma 7.1, employing a Hölder inequality in (7.5), and using (7.4), we find that

7.9 $$\begin{aligned} \frac{1}{2 \pi \textrm{i}} \oint _{\gamma } \textrm{e}^{\textrm{i}t z}\langle \psi _0, M_\lambda (z) \textrm{e}^{- \textrm{i}t H_0} \psi _0 \rangle \textrm{d}z = \textrm{e}^{- \textrm{i}\overline{m_0(E_0)} \lambda ^2 t} + \mathcal {O}(\mathcal {E})\,. \end{aligned}$$

Combining with (7.2) and taking the absolute value square of (7.9), we arrive at (2.21). This concludes the proof of Theorem 2.10. $\square$

## Data Availability

The authors declare that the data supporting the findings of this study are available within the paper.

## Additional proofs

### Upper bound on the renormalized shift: Perturbation argument for Lemma 4.6

The goal of this section is to prove the upper bound $|\Im \mathfrak {s}_0^{0,0}(E)| \lesssim \Delta ^2$, as claimed at the end of part (1) of the proof of Lemma 4.6 in Sect. 5.

This is done via a perturbative calculation, which we carry out in a slightly more general setting: Consider two spectral parameters $z_1=E_1-\textrm{i}0$, $z_2=E_2+\textrm{i}0$, such that $E_j$ is in the bulk of $\rho _j$, $j\in [2]$. Introducing the averaged and relative coordinates

$$\begin{aligned} D:=(D_1+D_2)/2,\quad z:=(E_1+E_2)/2+i0,\quad \Theta :=(D_2-D_1)/2-(E_2-E_1)/2\,, \end{aligned}$$

we find that $D_1-z_1=D-z-\Theta$ and $D_2-z_2=D-z+\Theta$. Let *M* be the solution of the MDE with the averaged coordinates, i.e.,

$$\begin{aligned} -\frac{1}{M}=z-D+\langle M\rangle . \end{aligned}$$

Using the identity $MM^* = \Im M/\langle \Im M\rangle$, it is easy to compute by Taylor expansion

A.1 $$\begin{aligned} M_2M_1= \frac{1}{\langle \Im M\rangle }\left( \Im M + 2\textrm{i}\Im \left[ \Im M\Theta M \right] +2\textrm{i}\Im \left[ \frac{\langle \Theta M^2\rangle }{1-\langle M^2\rangle }\Im M\cdot M \right] +\mathcal {O}( \vert \Theta \vert ^2)\right) \,, \end{aligned}$$

where $\mathcal {O}(|\Theta |^2)$ indicates terms containing at least two $\Theta$’s. Plugging (A.1) in the definition of the shift (4.12), we find that

$$\begin{aligned} \mathfrak {s}(z_1, z_2) + (z_2 - z_1) = - 2\frac{\langle \Theta \Im M\rangle + 2\textrm{i}\langle \Theta \Im \left[ \Im M\Theta M \right] \rangle +2\textrm{i}\left\langle \Theta \Im \left[ \frac{\langle \Theta M^2\rangle }{1-\langle M^2\rangle }\Im M\cdot M \right] \right\rangle +\mathcal {O}(\langle \vert \Theta \vert ^3\rangle )}{\langle \Im M\rangle + 2\textrm{i}\langle \Im \left[ \Im M\Theta M \right] \rangle +2\textrm{i}\left\langle \Im \left[ \frac{\langle \Theta M^2\rangle }{1-\langle M^2\rangle }\Im M\cdot M \right] \right\rangle +\mathcal {O}(\langle \vert \Theta \vert ^2\rangle )}. \end{aligned}$$

which implies

$$\begin{aligned} \begin{aligned}&\frac{\langle \Im M\rangle ^2}{2}[\mathfrak {s}(z_1, z_2) + (z_2 - z_1)] \\&= - \langle \Theta \Im M\rangle \langle \Im M\rangle -2\textrm{i}\langle \Im M\rangle \left\langle \left( \Theta -\frac{\langle \Theta \Im M\rangle }{\langle \Im M\rangle }\right) \Im M\Theta \Im M\right\rangle \\&-2\textrm{i}\langle \Im M\rangle \left\langle \left( \Theta -\frac{\langle \Theta \Im M\rangle }{\langle \Im M\rangle }\right) \Im \left[ \frac{\langle \Theta M^2\rangle }{1-\langle M^2\rangle }\Im M\cdot M \right] \right\rangle + \mathcal {O}(\langle |\Theta |^3\rangle ). \end{aligned} \end{aligned}$$

Using $\Im [z_2-z_1] = 0$ and $\Im [\langle \Theta \Im M\rangle \langle \Im M \rangle ] = 0$, the imaginary part is given by

$$\begin{aligned} \frac{\langle \Im M\rangle }{4}\Im \mathfrak {s}(z_1, z_2) = - \left\langle \left( \Theta -\frac{\langle \Theta \Im M\rangle }{\langle \Im M\rangle }\right) \Im M\left( \Theta \Im M+\Im \left[ \frac{\langle \Theta M^2\rangle }{1-\langle M^2\rangle } M \right] \right) \right\rangle + \mathcal {O}(\langle \vert \Theta \vert ^3\rangle ) \end{aligned}$$

and hence

$$\begin{aligned} \left| \Im \mathfrak {s}(z_1,z_2)\right|&= \frac{4}{\langle \Im M\rangle }\left| \left\langle \left( \Theta -\frac{\langle \Theta \Im M\rangle }{\langle \Im M\rangle }\right) \Im M\left( \Theta \Im M+\Im \left[ \frac{\langle \Theta M^2\rangle }{1-\langle M^2\rangle } M \right] \right) \right\rangle + \mathcal {O}(\langle |\Theta |^3\rangle )\right| \\&\lesssim \langle |\Theta |^2\rangle \,, \end{aligned}$$

since $\Vert \Theta \Vert \lesssim 1$. Specializing to the setting of Lemma 4.6 this result means that

$$\begin{aligned} \vert \Im \mathfrak {s}_0^{0,0}(E)\vert \lesssim \Delta ^2 + \vert f(E)-E\vert ^2 \lesssim \Delta ^2\,, \end{aligned}$$

where in the last step we used (4.13) and Lemma 4.5. This concludes the proof of the upper bound in part (1) of Lemma 4.6.

### The one-body stability operator is locally integrable

In Sect. 6, we frequently use that the one-body stability operator is locally integrable. This is the statement of the following lemma.

#### Lemma A.1

(Integral of one-body stability operator) Fix a (large) positive constant *L*. Uniformly in $\eta \in [0,1]$ and in *D* satisfying Assumption 2.2 with constant *L* we have

A.2 $$\begin{aligned} \int _{-L}^L \frac{\textrm{d}E}{\vert 1-\langle M^2(E+i\eta )\rangle \vert }\lesssim 1. \end{aligned}$$

#### Proof

With the notation (2.2), we use the classification of local minima of $\rho$ from [3, Theorem 7.1]. This result addresses the case of a *diagonal* deformation, while *D* in the formulation of Lemma A.1 does not need to be diagonal. Since the deterministic approximation *M*(*z*) to the resolvent $(H-z)^{-1}$ of a random matrix *H* depends only on the first two joint moments of entries of *H*, we have that *M*(*z*) in (A.2) coincides with the deterministic approximation to $(W_{\textrm{GUE}}+D-z)^{-1}$, where $W_{\textrm{GUE}}$ is a GUE matrix. Let *U* be a unitary diagonalizing *D*, i.e., $U^*DU=D_0$, where $D_0$ is diagonal. Invariance of GUE under unitary conjugations gives that $\widetilde{M}(z):=U^*M(z)U$ is a deterministic approximation to $(W_{\textrm{GUE}}+D_0-z)^{-1}$, so $\widetilde{M}(z)$ solves the MDE

$$\begin{aligned} -\widetilde{M}^{-1}(z)=z-D_0+\langle \widetilde{M}(z)\rangle ,\quad \Im z\Im \widetilde{M}(z)>0 \quad {\text {for}} \quad z\in \textbf{C}\setminus \textbf{R}\,. \end{aligned}$$

We remark that $\widetilde{M}$ satisfies the assumptions of [3, Theorem 7.1], since $\mathcal {S}=\langle \cdot \rangle$ is flat and by means of Assumption 2.2.

Thus, [3, Theorem 7.1] applied to $\widetilde{M}$ together with the observation $\langle \widetilde{M}\rangle =\langle M\rangle$ gives that there exist positive constants $\rho _*>0$ and $\delta _*>0$ dependent only on *L* such that for any local minimum $\tau _0$ of $\rho$ with $\rho (\tau _0)<\rho _*$ one of the following possibilities holds:

A.3a $$\begin{aligned}&\rho (\tau _0+\omega )\sim \min \lbrace \Delta ^{-1/6}\omega ^{1/2},\omega ^{1/3}\rbrace \,, \qquad  &   \omega \in [0,\delta _*]\,, \qquad  &   {\text {(left edge)}} \end{aligned}$$

A.3b $$\begin{aligned}&\rho (\tau _0+\omega )\sim \min \lbrace \Delta ^{-1/6}\vert \omega \vert ^{1/2},\vert \omega \vert ^{1/3}\rbrace , \qquad  &   \omega \in [-\delta _*,0]\,, \qquad  &   {\text {(right edge)}} \end{aligned}$$

A.3c $$\begin{aligned}&\rho (\tau _0+\omega )\sim \vert \omega \vert ^{1/3}, \quad  &   \omega \in [-\delta _*,\delta _*]\,, \qquad  &   {\text {(cusp)}} \end{aligned}$$

A.3d $$\begin{aligned}&\rho (\tau _0+\omega )\sim \tilde{\rho }+\min \lbrace \tilde{\rho }^{-5}\omega ^2,\vert \omega \vert ^{1/3}\rbrace ,\qquad  &   \omega \in [-\delta _*,\delta _*]\,, \qquad  &   {\text {(internal minimum)}} \end{aligned}$$

where $\tilde{\rho }\sim \rho (\tau _0)$ in (A.3d). In (A.3a), $\Delta :=1$ if $\tau _0$ is an extreme left edge of $\textrm{supp}\rho$ and $\Delta$ is the length of the gap between the intervals of support which ends at point $\tau _0$ otherwise (see also [3, Lemma 7.16]),^12^ for the right edge (A.3b) $\Delta$ is defined similarly.

As a first preparatory step for (A.2), we give a lower bound for $\vert 1-\langle M^2(E+\textrm{i}\eta )\rangle \vert$ in terms of $\rho (E)$. In fact, we will show that uniformly in $E\in [-L,L]$ it holds that

A.4 $$\begin{aligned} \vert 1-\langle M^2(E+\textrm{i}\eta )\rangle \vert \gtrsim \rho ^2(E)\,. \end{aligned}$$

By Lemma 5.1 the LHS of (A.4) has a lower bound of order $\rho ^2(E+\textrm{i}\eta ) + \eta /\rho (E+\textrm{i}\eta )$. Recall from [3, Proposition 2.4] that $\rho$ is 1/3-Hölder regular, i.e., there exists a constant $C_0$ depending only on *L* such that $\vert \rho (z_1)-\rho (z_2)\vert \le C_0\vert z_1-z_2\vert ^{1/3}$ uniformly in $z_1,z_2\in \textbf{C}$ with $\Im z_1 \Im z_2 > 0$ and $\vert z_j\vert \le 2\,L$, $j=1,2$. If $\eta \le \rho (E)^3/(2C_0)^3$, then $\rho (E+\textrm{i}\eta )\sim \rho (E)$, i.e., (A.4) holds. In the complementary case, $\eta > \rho (E)^3/(2C_0)^3$, we have $\rho (E+\textrm{i}\eta )<\rho (E)$ and hence $\eta /\rho (E+\textrm{i}\eta )\gtrsim \rho ^2(E)$, i.e., (A.4) again holds.

Now, armed with (A.4), we are ready to prove (A.2). We split the region of integration into several regimes according to the classification of local minima of $\rho$. For each local minimum $\tau _0$ with $\rho (\tau _0)<\rho _*$ the integration over $\tau _0+[-\delta _*,\delta _*]\cap \mathcal {D}$ will be considered separately. Here, $\mathcal {D}=\textbf{R}$ for cusps and internal minima, $\mathcal {D}=[0,+\infty )$ for left edges and $(-\infty ,0]$ for right edges. The set $\mathcal {D}$ is chosen in such a way that $\tau _0+[-\delta _*,\delta _*]\cap \mathcal {D}$ covers the part, where $\rho$ is positive and small. The complementary regimes, the *bulk regime* (where $\rho \ge \rho _*$) and the *gap regime* (where $\rho = 0$), are treated separately.

Bulk regime: It holds that $\rho (E)\ge \rho _*$, hence desired bound on the lhs. of (A.2) in the bulk regime immediately follows from (A.4).

Gap regime: Let $\tau _1<\tau _0$ be two edges of $\textrm{supp}\rho$ such that $\rho (E)=0$ for any $E\in [\tau _1,\tau _0]$; the cases when either $\tau _1$ is an extreme right edge or $\tau _0$ is an extreme left edge are treated similarly. Since $\partial _z M=M^2(1-\langle M^2\rangle )^{-1}$, we have $\vert 1-\langle M^2\rangle \vert ^{-1}\le 1 +\vert \langle M'\rangle \vert$. Together with (A.3a) and (A.3b), this gives that

$$\begin{aligned} \vert 1-\langle M^2(E+i\eta )\rangle \vert \gtrsim \left( \left( \min \lbrace {\vert E-\tau _1\vert , \vert E-\tau _0\vert \rbrace }\right) ^2+\eta ^2\right) ^{1/3}, \end{aligned}$$

so the integral of $\vert 1-\langle M^2(E+\textrm{i}\eta )\rangle \vert ^{-1}$ over $E\in [\tau _1,\tau _0]$ is uniformly bounded in $\eta \in [0,1]$.

$\underline{\text {Internal}\,\, \text {minimum}\,\, \text {with}\,\, \rho (\tau _0)<\rho _*:}$ Using (A.4) along with (A.3d), we find that

$$\begin{aligned} \int _{-\delta _*}^{\delta _*} \frac{\textrm{d}\omega }{\vert 1-\langle M^2(\tau _0+\omega +i\eta )\rangle \vert }\lesssim \int _{-\delta _*}^{\delta _*} \frac{\textrm{d}\omega }{\rho ^2(\tau _0+\omega )}\lesssim \int _0^{\tilde{\rho }^3} \frac{\textrm{d}\omega }{\tilde{\rho }^2}+\int _{\tilde{\rho }^3}^{\delta _*}\frac{\textrm{d}\omega }{\omega ^{2/3}}\lesssim 1 \,. \end{aligned}$$

Cusp regime: This works in the exact same way as the internal minimum, using (A.3c) instead of (A.3d).

Edge regime: Let $\tau _0$ be a left edge of $\rho$, for the right edge the argument is the same. First, [3, Corollary 5.3] gives that $\vert 1-\langle M^2(z)\rangle \vert \gtrsim \rho (z)(\vert \sigma (z)\vert +\rho (z))$, where $\sigma (z)$ is a 1/3-Hölder regular function in $\{ z \in \textbf{C}: \Im z > 0\}$ (by [3, Lemma 5.5]) and $\vert \sigma (\tau _0)\vert \sim \Delta ^{1/3}$ (by [3, Theorem 7.7, Lemma 7.16]). Therefore, there exists a (small) positive constant $c\sim 1$ such that for all *z* with $\Re z\in [\tau _0, \tau _0+c\Delta ]$ and $\Im z\in [0,c\Delta ]$ it holds that $\vert \sigma (z)\vert \sim \Delta ^{1/3}$. It is easy to see that the integral of the one-body stability operator over $[\tau _0+c\Delta ,\tau _0+\delta _*]$ has an upper bound of order one by means of (A.4) and (A.3a). In the complementary regime $[\tau _0,\tau _0+c\Delta ]$ we distinguish between two cases (i) $\eta \in [0,c\Delta ]$ and (ii) $\eta >c\Delta$. In the first case, note that, by the integral representation $\rho (E+i\eta )=\int _{\textbf{R}} \textrm{d}x \rho (x) \eta /((x-E)^2+\eta ^2)$ and (A.3a), it holds that $\rho (E+\textrm{i}\eta )\gtrsim \rho (E)$ for $E\in [\tau _0,\tau _0+c\Delta ]$. Thus,

$$\begin{aligned} \int _0^{c\Delta }\frac{\textrm{d}\omega }{\vert 1-\langle M^2(\tau _0+\omega +i\eta )\rangle \vert }\lesssim \Delta ^{1/3}\int _0^{c\Delta }\frac{\textrm{d}\omega }{\omega ^{1/2}(\Delta ^{1/2}+\omega ^{1/2})}\lesssim 1. \end{aligned}$$

In the second case, $\eta >c\Delta$, we use (A.4) and the bound $\vert 1-\langle M^2(z)\rangle \vert \gtrsim |\Im z|$ to get

$$\begin{aligned} \int _{0}^{c\Delta }\frac{\textrm{d}\omega }{\vert 1-\langle M^2(\tau _0+\omega +i\eta )\rangle \vert } \lesssim \int _{0}^{c\Delta }\frac{\textrm{d}\omega }{\rho ^2(\tau _0+\omega )+\Delta }\sim \Delta ^{1/3}\int _0^{c\Delta }\frac{\textrm{d}\omega }{\omega + \Delta ^{4/3}}\lesssim 1\,, \end{aligned}$$

which concludes the proof for the regular edge.

A careful examination of the proof shows that all implicit constants in the inequalities above depend only on *L*. $\square$

## Footnotes

1 This means that when writing $\psi _0 = \sum _n c_n^{(i)} \phi _n^{(i)}$ in the eigenbasis $\{\phi _n^{(i)}\}_n$ of $H_i$, only coefficients $c_n^{(i)}$ corresponding to an eigenvalue close to $E_0$ are non-vanishing.

2 In the very extreme case, when the difference $H_1 - H_2$ is small compared to the local eigenvalue spacing one observes Gaussian instead of exponential decay, $\mathfrak {M}(t) \approx \textrm{e}^{-\gamma t^2}$ (see, e.g., [29, Section 2.3.1])

3 Within Theorem 2.10, the Hamiltonians $H_1 = D$ and $H_2 = D + \lambda W$ will be denoted by $H_0$ and $H_\lambda$, respectively.

4 A careful examination of our proof reveals that the entries of *W* need not be distributed identically. Indeed, only the matching of the second moments is necessary, but higher moments can differ.

5 In [1–3], the scDos has been thoroughly analyzed in increasing generality of the ensemble. It is supported on finitely many finite intervals and, roughly speaking, there are three different regimes for the behavior or $\rho$: In the *bulk* $\rho$ is strictly positive; at the *edge*, $\rho$ vanishes like a square root at the edges of every supporting interval which are well separated; at the *cusp*, where two intervals of support (almost) meet, $\rho$ behaves (almost) as a cubic root. Correspondingly, $\rho$ is locally real analytic, Hölder-1/2, or Hölder-1/3 continuous, respectively. Near the singularities, it has an approximately universal shape (see (A.3a)–(A.3d) in the proof of Lemma A.1).

6 If $D_1$ or $D_2$ had a nonzero trace, it could be absorbed by a simple (scalar) energy shift.

7 To be precise, when $\delta < 1$, we use the slightly improved average *global* law $|\langle A(W-z)^{-1} \rangle - m(z) \langle A \rangle | \prec \delta ^2 \Vert A \Vert /N$ (see, e.g., [21, Theorem 2.1]).

8 Here, $C^{1,1}(J)$ denotes the set of continuously differentiable functions with a Lipschitz-continuous derivative on an interval *J*, equipped with the norm $\Vert f\Vert _{C^{1,1}(J)}:= \Vert f\Vert _{C^1(J)} + \sup \limits _{x,y\in J: x\ne y} \frac{|f'(x)-f'(y)|}{|x-y|}$.

9 In order to guarantee convergence of the BCH expansion (3.4), we need the time *t* to be small enough such that $|t|(\Vert H_1 \Vert + \Vert H_2 \Vert ) < \log 2$ [46], which can be achieved in an open interval around zero, since $\Vert D_i\Vert \lesssim 1$ and $\Vert W \Vert \le 2 + \epsilon$ with very high probability.

10 By carefully tracking $\delta$ throughout the proof, one can see that the dependence is inverse polynomially, $C(\delta ) \lesssim \delta ^{-n}$ for some $n \in \textbf{N}$. This will, however, be completely irrelevant for our purposes.

11 In view of (4.10), note that the supports $\textrm{supp}(\rho _1), \textrm{supp}(\rho _2)$ of the scDos of $H_1$ and $H_2$ are contained in $[-(L+2), (L+2)]$.
